# Electronic data collection, management and analysis tools used for outbreak response in low- and middle-income countries: a systematic review and stakeholder survey

**DOI:** 10.1186/s12889-021-11790-w

**Published:** 2021-09-25

**Authors:** Patrick Keating, Jillian Murray, Karl Schenkel, Laura Merson, Anna Seale

**Affiliations:** 1 London School of Hygiene and Tropical Medicine, London, UK; 2 United Kingdom Public Health Rapid Support Team, London, UK; 3 World Health Organisation, Geneva, Switzerland; 4 University of Oxford, Oxford, UK

**Keywords:** Data collection, Data analysis, Data management, Outbreaks, Low income, Middle income

## Abstract

**Background:**

Use of electronic data collection, management and analysis tools to support outbreak response is limited, especially in low income countries. This can hamper timely decision-making during outbreak response. Identifying available tools and assessing their functions in the context of outbreak response would support appropriate selection and use, and likely more timely data-driven decision-making during outbreaks.

**Methods:**

We conducted a systematic review and a stakeholder survey of the Global Outbreak Alert and Response Network and other partners to identify and describe the use of, and technical characteristics of, electronic data tools used for outbreak response in low- and middle-income countries. Databases included were MEDLINE, EMBASE, Global Health, Web of Science and CINAHL with publications related to tools for outbreak response included from January 2010–May 2020. Software tool websites of identified tools were also reviewed. Inclusion and exclusion criteria were applied and counts, and proportions of data obtained from the review or stakeholder survey were calculated.

**Results:**

We identified 75 electronic tools including for data collection (33/75), management (13/75) and analysis (49/75) based on data from the review and survey. Twenty-eight tools integrated all three functionalities upon collection of additional information from the tool developer websites. The majority were open source, capable of offline data collection and data visualisation. EpiInfo, KoBoCollect and Open Data Kit had the broadest use, including for health promotion, infection prevention and control, and surveillance data capture. Survey participants highlighted harmonisation of data tools as a key challenge in outbreaks and the need for preparedness through training front-line responders on data tools. In partnership with the Global Health Network, we created an online interactive decision-making tool using data derived from the survey and review.

**Conclusions:**

Many electronic tools are available for data -collection, −management and -analysis in outbreak response, but appropriate tool selection depends on knowledge of tools’ functionalities and capabilities. The online decision-making tool created to assist selection of the most appropriate tool(s) for outbreak response helps by matching requirements with functionality. Applying the tool together with harmonisation of data formats, and training of front-line responders outside of epidemic periods can support more timely data-driven decision making in outbreaks.

**Supplementary Information:**

The online version contains supplementary material available at 10.1186/s12889-021-11790-w.

## Introduction

Infectious disease outbreaks pose a serious global health challenge, as illustrated by the worldwide pandemic of COVID-19. In the last decade alone, the World Health Organization (WHO) has declared six Public Health Emergency of International Concern (PHEIC) associated with infectious diseases [1–3]. Low and middle-income countries (LMICs) with weaker health systems are particularly at risk with limited surveillance leading to late detection and response to outbreaks [4, 5]. During outbreaks, timely collection, management, sharing, analysis and reporting of data are required to ensure that interventions are appropriately targeted and effective in supporting outbreak control.

The use of information and communication technologies (ICT) for health - eHealth, is growing in importance as it facilitates access to and delivery of health services as well as collection, management and analysis of infectious and non-communicable disease data [6, 7]. Electronic tools and solutions offer many advantages over traditional paper-based data collection, including more rapid data collection and transfer, use of checks/validation to improve data accuracy, and collection of more diverse data types including images, audio and barcodes. They can provide cost savings and are more environmentally friendly [8–11]. However, electronic tools also have limitations in some contexts, for example if there are requirements for stable electricity, internet or phone connectivity.

Electronic tools can also be used during outbreaks to support timely collection and analysis of data. However, in practice, in outbreak response we have observed data are often collected on paper and day-to-day programs for analysis being used, foregoing tools better designed to meet needs. There are a growing number of tools available for use in outbreaks and emergencies [12, 13]. A recent review identified 58 mobile tools developed for, or used during, the Ebola outbreak in West Africa in 2013–2016 [12]**.** There are however challenges to the use of electronic tools in outbreaks and emergencies. Emergencies require rapid deployment of frontline workers for data collection, and while there are now many electronic tools to support this, various similar tools for data collection may occur during the same outbreak at the same time in a fragmented manner, due to lack of coordination. The number of similar tools combined with the compressed timeframes can lead to little time for deliberation over optimal data collection methods. There are also requirements in these contexts - emergencies and outbreaks in LMICs often occur in settings with the most limited infrastructures and tools in these settings need to be flexible to function in these environments. They also need to be able to be incorporated into existing data or surveillance infrastructures and allow for interoperability and/or data sharing between organisations. Ideally, these electronic tools should be employed both for routine surveillance activities and outbreak response in an integrated, sustainable approach.

Choosing the most appropriate electronic tool for an outbreak response is important, but it is also essential to harmonise data collection and ensure interoperability. Some projects seek to support harmonisation of how we collect data independently of the tool we use, including the Humanitarian Exchange Language (HXL) and the WHO “Outbreak Toolkit” [14, 15]. Standardising the choice, format and/or naming/tagging of the data variables collected between and within organisations during outbreaks and humanitarian emergencies (such as through standardised case investigation forms, unique agreed reporting categories, standardised data formats and data dictionaries) facilitates more efficient data sharing and timelier data analyses for rapid decision making.

In outbreaks and emergencies, diverse technical characteristics are required for an electronic data system to support the most effective outbreak response. With limited time and resources, it is unsurprising that decision makers may have difficulty identifying the most appropriate tools for their needs. Here we aim to identify and describe characteristics of electronic data collection, management and analysis tools used for infectious disease outbreaks in LMICs, the functionalities most commonly required in outbreaks, in order to facilitate decision making on appropriate tool selection. We also aimed to make this information more easily accessible to relevant stakeholders through the creation of an interactive and dynamic online decision-making tool.

## Methods

### Search strategy

We followed PRISMA guidelines to conduct the systematic review [16]. We searched five electronic databases: MEDLINE, EMBASE, Global Health, Web of Science and CINAHL. The search terms included four categories: LMICs, outbreaks or epidemics or early warning alert and response or humanitarian emergencies, data management, collection and analysis and electronic tools. Search terms are listed in additional file 1 for all databases. The search strategies included indexed terms where possible. LMIC filters based on World Bank categorizations were used from Ovid [17].

### Inclusion and exclusion criteria

We included studies if they described electronic data collection, management or analysis tools that were used in low- and middle-income countries either for detection or response to an infectious disease outbreak (animal or human) alone or as part of a humanitarian emergency, or as part of an early warning and alert system. In addition, studies that analysed outbreak data or performed operational disease modelling analyses were included. Studies published from 1st January 2010 to 12th October 2018 in English, French, German, Portuguese or Spanish were included. An update of the original search was conducted which included studies published from October 2018 to May 2020.

We excluded studies that described non-communicable diseases or drug epidemics, clinical trials in outbreaks; seroprevalence studies; qualitative studies, unless directly linked to data tools for collection, management and analysis of outbreak or outbreak-related humanitarian data. Studies that focused on retrospective analysis of infectious disease surveillance data to identify outbreaks as well as articles describing electronic data tools without any operational application to outbreak response were also excluded. Similarly, modelling analyses based on surveillance or simulated data as well as tools for humanitarian response or preparedness without any link to outbreak response were excluded. Hospital information management tools, unless specifically developed for an outbreak, were also excluded. Review articles and studies for which full text articles were not available open access were also excluded.

### Identification of potentially eligible studies

We exported identified studies to Endnote (version 8, Clarivate Analytics, Philadelphia, USA) and removed any duplicates. Two authors (PK and JM) independently assessed the relevance of all titles and abstracts based on inclusion and exclusion criteria. In the case of differing views on the inclusion of an article, consensus was reached by discussion between the two researchers. Full-text articles were retrieved for all potentially relevant studies. Two authors (PK and JM) independently assessed the full text articles using the inclusion and exclusion criteria.

### Data extraction

We extracted information, using a standardised form, on the electronic tools from the included articles examining cost, license type, compatibility with windows, mac and linux, location of data storage, ability to visualise data, whether data could be collected with a mobile application (also the type of mobile devices that could be used for data collection) or via a web interface, data encryption functionality, collection of GPS data, and ability to collect data offline (see additional file 2 for the definitions used for the data extraction). We supplemented data from the systematic review with additional detail on technical capacities and overall functions either via direct contact with the electronic tool developers and/or reading information provided in their websites.

### Stakeholder survey

To identify electronic tools used in recent outbreaks in LMICs, we invited key stakeholders, such as Global Outbreak Alert and Response Network (GOARN) members which includes National Public Health Institutes, Ministries of Health, United Nations Agencies, National and international laboratory teams, non-governmental organisations (e.g. Doctors Without Borders/Médecins Sans Frontières), Training Programs in Epidemiology and Public Health Interventions Network (TEPHINET) and related Field Epidemiology Training Programmes to participate in an online survey (created using Enketo and available in English and French) in May 2019 (see additional file 3 for the data dictionary of the survey). To be eligible to complete the survey, participants needed to have responded to outbreaks in World Health Organisation (WHO) grade two or three priority countries that included Afghanistan, Bangladesh, Cameroon, Central African Republic, Chad, Democratic Republic of Congo, Ethiopia, Iran, Iraq, Mali, Nigeria, Occupied Palestinian Territory, Pakistan, Somalia, South Sudan, Sudan, Syrian Arab Republic, Turkey/North Syria, Ukraine and Yemen. We selected these countries to identify tools that had been used in recent outbreaks during acute or protracted emergencies. We asked participants to identify the electronic data collection, management and analysis tools used during the outbreaks and to specify what they used the tools for. We focused on key pillars of outbreak response and asked participants to specify which electronic tools they used to collect/manage and analyse alert, case investigation, contact tracing, health promotion, case management, infection, prevention and control, laboratory, surveillance, clinical trial and water and sanitation data. We also asked participants their reasons for selecting the specific tool, and for any suggestions on how to improve the tools. In addition, participants were asked to rank and comment on data collection challenges in outbreaks.

### Data analysis

Counts and proportions of data obtained either from the systematic review or the stakeholder survey were calculated using R version 3.6.1, R Foundation for Statistical Learning, Vienna, Austria. Text data from the stakeholder survey were analysed by reviewing responses and identifying the most frequently shared views.

## Results

### Identification of electronic tools - systematic review and stakeholder survey

**Fig. 1:**
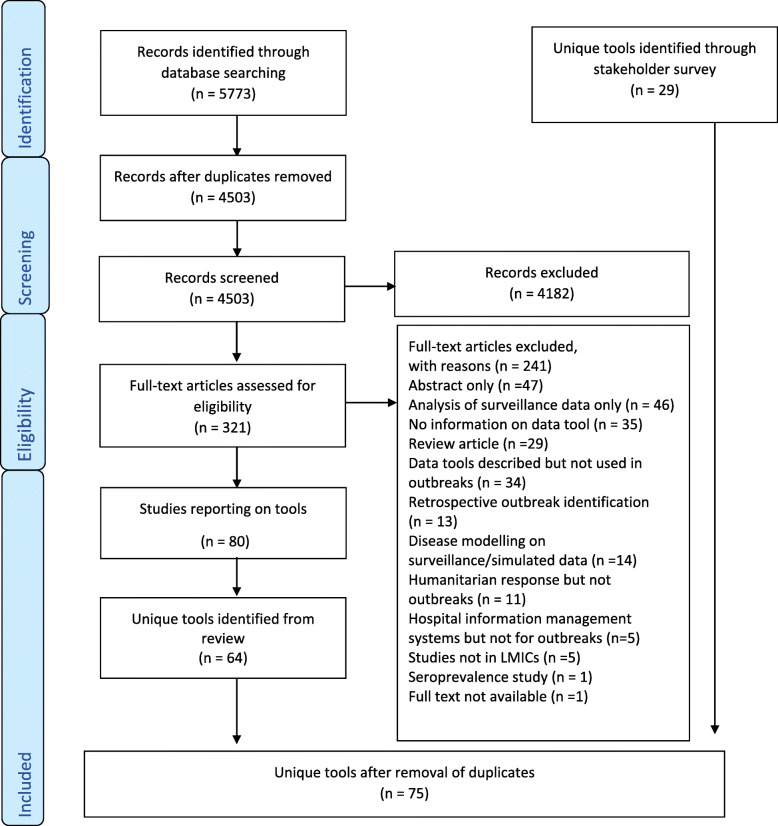
PRISMA flow chart for systematic literature review (2010–2020) [16]

We identified 5773 studies from the five selected databases across the period 2010–2020 and after deduplication, 4503 potentially relevant studies remained. We screened full-text articles for 321 studies, of which 80 were included [11, 18–96] (see additional file 4 for a list of excluded studies and the reasons for exclusion). From 80 studies included, we identified 64 unique electronic data tools (Fig. 1). Of these 80 studies, twelve, eleven, five and three studies were focused on Ebola, Dengue, Cholera and COVID-19, respectively. Four and six studies described animal outbreaks and early warning systems, respectively. Six custom-made applications were also among the included studies. Among the 64 tools reported, 12 were identified through the update to the systematic review from 2018 to 2020. No major differences were observed in terms of the types of tools (i.e. similar proportions of data collection, management and analysis tools were found in both periods) identified in the periods 2010–2018 and 2018 and 2020.

Fifty-five individuals consented to complete the online stakeholder survey, of which 41 were eligible for inclusion. Four Ministries of Health and sixteen organisations were represented, of which Doctors Without Borders and the WHO made up 22% (*n* = 9) each of the eligible survey respondents (see additional file 5 for a summary of all organisations that participated). The Democratic Republic of Congo (*n* = 20), Nigeria (*n* = 11) and Bangladesh (*n* = 10) were the countries most frequently mentioned for outbreak response by respondents. However, respondents had been involved in outbreak response in 17 out of the 20 listed countries. Epidemiologists represented the majority of survey respondents (59%, *n* = 24). Respondents reported working at international, national, regional and field levels, with the majority (51%, *n* = 21) working at field level. Twenty-nine unique tools were reported in the stakeholder survey and after deduplication with the tools identified from the systematic review, 75 tools were included in this study for further characterisation (Fig. 1).

### Uses of electronic data tools

The 75 identified tools reported in the systematic review and survey covered data collection [33], management [13] and analysis [49] activities as detailed in Table 1. Of note, many of the tools could be used for collection and/or management and/or analysis and almost half of the analysis tools related to analysis/visualisation of sequencing data. In the following paragraphs the proportion of data collection, management and analysis tools in terms of their uses and other characteristics is calculated, but these calculations exclude tools for which it was not possible to ascertain if the tool had the specific use/characteristic or not. For data collection tools, surveillance was the most frequently reported use (81%, *n* = 25), followed by case management and health promotion (69% and 54%, respectively). Electronic data collection tools were less frequently reported as being used for collection of infection, prevention and control data (25%, *n* = 6) and for WASH assessments (30%, *n* = 7). For data management, the majority of the thirteen reported data management tools allowed data cleaning, survey generation and more general data management functionality. For data analysis, of the 49 identified data analysis tools, data visualisation (72%, *n* = 21) and mapping (69%, *n* = 18) were the most commonly reported uses and phylogenetic analysis the least (43%, *n* = 13).

**Table 1:** Uses and number of electronic data collection tools as reported in the review or survey for outbreak response in LMICs

Purpose of tool	Use	Yes (n)	No (n)	Unknown (n)	% Yes^a^
Data collection (*n* = 33)	Surveillance	25	6	2	81
	Case management	18	8	7	69
	Other collection activities	13	8	12	62
	Health promotion	13	11	9	54
	Laboratory	13	12	8	52
	Case investigation	13	13	7	50
	Alerts	11	13	9	46
	Contact tracing	11	14	8	44
	WASH	7	16	10	30
	IPC	6	18	9	25
Data management (n = 13)	Data management	10	0	3	100
	Data cleaning	8	1	4	88
	Survey generation	10	2	1	83
	Other data management activities	7	3	3	70
Data analysis (*n* = 49)	Data visualisation	21	8	20	72
	Mapping	18	8	23	69
	Descriptive analysis	16	8	25	67
	Data reporting	15	8	26	65
	Data cleaning	11	8	30	58
	Dashboard creation	12	9	28	57
	Spatial analysis	12	10	27	55
	Other data analysis activities	10	9	30	53
	Modelling	11	10	28	52
	Phylogenetic analysis	13	17	19	43

^a^The denominator is the sum of yes and no

**Fig. 2:**
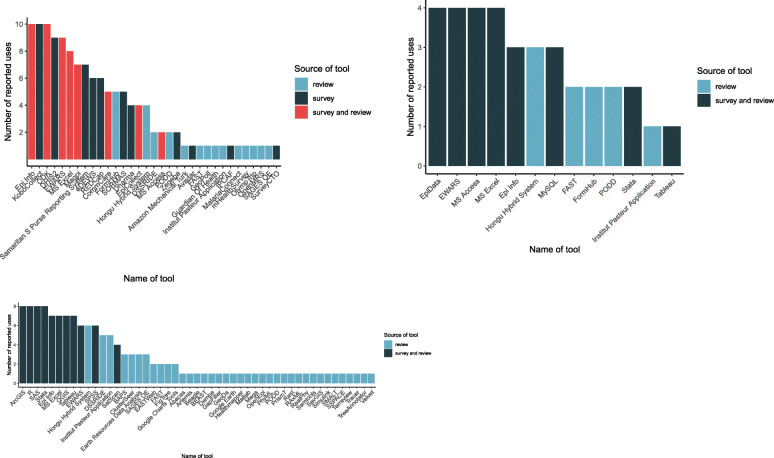
Reported number of uses per electronic tool as determined by the stakeholder survey and systematic review: (a) data collection (b) data management and (c) data analysis tools

The reported uses of electronic data collection, management and analysis tools in outbreak response identified in the systematic review and stakeholder survey are given in Fig. 2a, b and c for each tool respectively. Epi Info, KoBoCollect and ODK were the tools most widely reported in use to collect a range of outbreak data, followed closely by DHIS2 and EWARS (Fig. 2a). There were fewer differences across reported data management tools and MS Access, Excel, EWARS and Epi Data offered the same number of functionalities (Fig. 2b). Among data analysis tools, ArcGIS, R, SAS and Stata were reported as being used with the greatest diversity of functions (Fig. 2c). See additional file 6 for a summary of the tools that organisations with at least two respondents reported. The number of uses identified per tool varied depending on whether the tool was reported in the survey and/or the systematic review, such that tools reported in the systematic review only generally had fewer reported uses (see additional file 7 for a breakdown of where tools were identified from, the period of the systematic review and their reported uses).

### Characteristics of electronic data tools

With further investigation via contacting tool developers or through the tool websites, we found that many of the tools identified in the review and stakeholder had more functionalities and uses compared to data obtained from the survey and review alone. Twenty-eight out of the 75 identified tools could perform data collection, management and analysis functions to a greater or lesser extent in comparison to seven tools based on data only available in the review and survey. The 28 tools that incorporate data collection, management and analysis functionalities (Table 2) have overlapping characteristics, with 90% (*n* = 18) of tools supporting data encryption, 88% (*n* = 23) offering data visualisation and 85% (*n* = 22) providing web data entry functionalities. In addition, we found the majority of tools were compatible with Windows (100%) and Apple (85%). However, only 63% (*n* = 15) are open source software and 87% (*n* = 20) of the software are free (see additional file 8 for more detailed information per tool).

**Table 2:** Characteristics of tools that have data collection, management and analysis functionalities based on systematic review, survey and website/contact with tool developers (*n* = 28)

Characteristics	Yes (n)	No (n)	Unknown (n)	% Yes^a^
Windows compatible	26	0	2	100
Data encryption	18	2	8	90
Linux compatible	15	2	11	88
Data visualisation	23	3	2	88
Free software	20	3	5	87
Apple compatible	17	3	8	85
Offline data collection	22	4	2	85
Web data entry available	22	4	2	85
GPS collection	20	4	4	83
Mobile application available	21	7	0	75
Open source	15	9	4	63
Cloud and local server for data storage	14	14	0	50
Two or more mobile operating systems supported	13	15	0	46

^a^The denominator is the sum of yes and no

### Factors influencing tool selection

Survey participants were asked an open question on what influenced their choice of tool, and 10 factors emerged as key influencers: [1] cost [2]; user friendliness [3]; speed to configure data collection forms/deploy the system [4]; availability of the tool [5]; previous experience with a tool either by individuals, within specific organisations or by Ministry of Health personnel [6]; open source tool [7]; offline use [8]; availability of specific functionalities e.g. audit log [9]; if the tool was viewed as the “sector standard” and [10] presence of a strong userbase/community of support.

### Improving currently available tools

Survey participants were also asked another open question to comment on how their currently used electronic tools could be improved. Ten improvements were recommended: [1] reduce the cost [2]; improve the interoperability [3]; improve user-friendliness [4]; increase the flexibility and customisability of the tool, which would reduce the reliance of local staff on centralised teams/deployment of specialists [5]; provide more training resources and in more languages, with a suggestion to build training modules within the tools [6]; simplify the local hosting procedures [7]; improve data visualisations [8]; allow more advanced analyses to be performed [9]; improve multiple user management and [10] improve support for longitudinal data.

### Data collection challenges in outbreaks

Survey respondents’ were asked to rank nine potential data collection challenges during outbreaks in LMICs, which were based on the experiences of the authors: [1] ensuring data confidentiality [2]; harmonisation of data tools [3]; operating in insecure environments [4] lack of or limited internet access [5]; language barriers [6]; physical access to populations/areas [7]; lack of or limited power supply [8]; community resistance and [9] lack of or limited telecommunications/mobile connectivity. Based on the median ranking score, respondents rated harmonisation of data tools as the most difficult challenge of those listed, but there was overlap with other challenges including operating in insecure environments and lack of or limited internet access. Respondents were also asked to comment on other data collection challenges and provide any further reflections on electronic tools and highlighted the following: [1] integrated electronic tools should be capable of doing all the basics for outbreak response with minimal training [2]; there is a need for greater coordination of responding organisations including around tool/template and data sharing [3]; there is insufficient training of data collectors including on how the data collected would be used [4]; the need to provide training on electronic tools outside of outbreak periods and to focus on getting the basics right and [5] feedback loop ensuring that data collection teams can see or access the results/analyses.

## Discussion

Capturing accurate, timely data on outbreaks is central to any outbreak response and highlighted further during the COVID-19 pandemic, as it allows timely decision making on response activities, whether localised or worldwide. The COVID-19 pandemic alone has led to the development of a number of new tools for contact tracing and other purposes used across multiple settings, although primarily in high-income countries, but also raised concerns about data protection/security [97]. We identified a wide range of electronic tools to support data collection, management and analysis in outbreak response. Key requirements for tools selected at present include availability and whether training is needed, as well as cost, and if the software is open source. This may lead to use of tools without the optimal functionalities for outbreak response as most members of an outbreak response team, including non-analytical staff, are familiar with standard software. Specifically designed and integrated tools, can better support outbreak investigation across data collection, management and analysis. With more advanced analytics being incorporated into outbreak response and with data management being an integral part of data analysis, ease of use should not drive choice [98]. Analytic tools used in an emergency should ideally be part of routinely used surveillance software or at least be compatible with and complement such routine surveillance software. In addition, analytic tools should also reflect the analysis and data management required for outbreak control. Supporting decision makers to consider and match the functionalities of a tool, with their requirements, is a key part of enabling optimal use of tools, as well as overcoming practical constraints through preparedness, to support training and implementation.

We identified electronic tools in the published literature as well as tools organisations recently used in outbreak response in LMICs. However, the diversity of information may be limited by the responses to the survey, which was sent out to GOARN members (200 technical institutes and over 600 partners worldwide), with only 20 organisations (four Ministries of Health from LMICs) completing the survey [99]. In addition, the majority of respondents were epidemiologists from international organisations including MSF and the WHO and thus the results may not fully reflect the electronic tools being used across different pillars in outbreak response nor those used routinely in LMICs for outbreaks in which international organisations do not get involved. The survey did, however, support identification of tools in the grey literature, which also formed a key part of the findings of a recent review on tools used in the Ebola outbreak [12]. In addition, the period covered by this study included the first months of the COVID-19 pandemic but we only identified one novel tool developed to support COVID-19 response [25]. This tool called the Honghu Hybrid System incorporated clinical, laboratory and social media platform data to help COVID-19 surveillance and control. It is likely that the Honghu Hybrid System is one of many such tools that have been developed since the start of the pandemic and a COVID-19 specific review would enable the identification of further such innovations.

Despite these limitations, we identified a wide range of tools, and showed that tools which are open source are important, as well as training to support their use. Outbreak response personnel, especially front-line workers, must be trained in the use of integrated tools that can perform key basic analyses to support rapid decision making. Training on such tools during non-emergency periods is thus vital. Training initiatives such as the DHIS2 academy enable individuals to build capacity in the implementation of DHIS2 at national and higher levels outside periods of outbreaks/emergencies [100]. Similar training initiatives could be used to strengthen capacity of front-line workers on other outbreak deployment tools and factors such as DHIS2-compatibility, being open source and having large and diverse community user bases could help narrow the scope of tools for such initiatives. Moreover, it would enable the identification of tools more likely to be maintained and developed compared to the use of more niche tools. It was surprising to find a small number of tools created for specific projects such as the AVADAR, mHealthSurvey, OlympTRIP and the Institut Pasteur applications [57, 59, 85, 101]. Through investment in existing open-source tools, researchers and Ministries of Health could add new functionalities to these tools benefitting larger user communities and limiting the proliferation of electronic tools with similar functionalities. In addition, inclusion of regularly updated training modules (in multiple languages) within the tools themselves could offer opportunities to strengthen capacity of front-line workers during non-emergency periods.

The large number of tools for data collection, management and analysis, with similar functionalities (and limited economic evaluation) makes it difficult for decision makers to select the most appropriate tool for their needs. This range of tools has both advantages and disadvantages, as no one tool may be suitable for all types of outbreaks across multiple different contexts. Therefore, decision makers would benefit from support to match the functionalities of tools with their specific requirements and contexts. To address this point, from this study, we created an online decision-making tool (https://uk-phrst.tghn.org/tools-platforms/tools/data-tool-finder-app/) based on data collected from this study to support organisations to identify the most appropriate tool(s) for their needs (based on data in additional file 8). The online decision-making tool enables users to select electronic tools based on their requirements, rate tools they have already used and to make suggestions of any extra tools to include in the decision-making tool. This will allow it to both identify better performing tools and to remain up to date with electronic tools being used and developed for outbreak response in LMICs. Users can take into consideration factors such as the cost, the license type, availability of a mobile application, ability to perform data visualisation and location where data are hosted.

The need for better data harmonisation and improved interoperability between tools was another key finding of this study. Improving the interoperability and harmonisation between outbreak data collection tools and broader Health Information Systems facilitating routine surveillance activities (such as DHIS2) is important. Harmonising use of data tools within and between organisations, supporting training and improving interoperability remain key to improving uptake and use of electronic tools in outbreak response in LMICs. Harmonising electronic tool use across regions would allow for the roll out of standardised training to regional and national rapid response teams and thus improve their ability to support each other during outbreaks. The current COVID-19 pandemic illustrates this further and that it is not just in LMICs where harmonisation of tools and training is required but it would be highly beneficial in all countries. All pillars of the COVID-19 response rely on data to inform interventions/actions taken from case management, contact tracing, risk communication to infection prevention and control and a harmonisation of data formats, tools and training would save countries time and financial resources.

Improving the consistency of the choice and format of data collected in emergencies is recognised internationally [14, 15]. Using the same data templates would greatly facilitate management, analysis and sharing of data and thus more rapid and better-informed decision making. Enabling more rapid sharing of data during a pandemic such as COVID-19 within and between countries would further support more rapid and appropriate decision making. In addition, ensuring that data collectors know why they are collecting the data, how it will be used and have access to the results are all important in maintaining motivation to work in challenging outbreak environments.

In conclusion, a multi-faceted approach that considers both the type and format of data being collected, the use of integrated electronic tools that ideally function both for routine surveillance and outbreak response as well as a focus on training and ongoing capacity strengthening on data collection, management and analyses is needed to address this most urgent challenge.

## Supplementary Information

**Additional file 1.** Search strategies used in Medline, Embase and Global Health, CINAHL and Web of Sciences databases. This file contains three sets of search strategies (1) those used for OVID database – Medline, Embase and Global Health; (2) CINAHL database and (3) Web of Sciences database.

**Additional file 2.** Definitions used for data extraction. Table shows terms used for technical characteristics of electronic tools examined and their definitions.

**Additional file 3.** Data dictionary of the online Enketo Stakeholder survey on electronic data collection, management and analysis tools. The data dictionary shows the variable names, question text, response options/question type, skip logic and validation information as well as whether a question was required or not.

**Additional file 4.** List of studies excluded from the systematic review (2010–2020) and their reasons for exclusion. Dataset that describes the studies excluded from the systematic review (2010–2020) and includes the first author, title, journal and year of publication of the study and the reason for exclusion.

**Additional file 5.** Number and percentage of respondents to the stakeholder survey per responding organisation. Table shows the number and percentages of respondents per organisation that responded to the stakeholder survey.

**Additional file 6.** List of electronic tools reported by organisations with at least two respondents to the survey. Table shows number of stakeholder survey respondents per organisation (where at least 2 respondents from the same organisation responded) and the data collection, management and analysis tools used per organisation.

**Additional file 7.** List of electronic tools identified and their reported uses from the systematic review (2010–2020)/stakeholder survey. Dataset that describes the reported uses of the electronic tools either from the systematic review (2010–2020) and/or from the stakeholder survey. Where no data were found on a particular use of a tool, “don’t know” was entered in the database and where a tool only had one function (data collection or management or analysis), “NA” for not applicable was added to the relevant columns.

**Additional file 8.** Technical characteristics of tools as identified from the review, survey, and tool developers’ websites/direct contact. Dataset that describes the technical characteristics of the electronic tools as identified from the systematic review (2010–2020), survey or from review of software websites or contact with software developers. Where no data were found on a particular characteristic of a tool, “don’t know” was entered in the database and where a tool only had one function (data collection or management or analysis), “NA” for not applicable was added to the relevant columns. The Samaritan’s Purse Reporting System was excluded from this database on request from the organisation.

**Additional file 9.** PRISMA checklist. PRISMA checklist describes how PRISMA criteria were applied to the conduct of the systematic review.

## Data Availability

The datasets created as part of this study are available from the corresponding author on request.
