# Supplementary material for: Electronic data collection, management and analysis tools used for outbreak response in low- and middle-income countries: a systematic review and stakeholder survey
Source: BMC Public Health. 2021 Sep 25;21:1741. doi: 10.1186/s12889-021-11790-w (PMC8464108; doi:10.1186/s12889-021-11790-w)
Supplement: Supplementary file 1 — Additional file 1. Search strategies used in Medline, Embase and Global Health, CINAHL and Web of Sciences databases. This file contains three sets of search strategies (1) those used for OVID database – Medline, Embase and Global Health; (2) CINAHL database and (3) Web of Sciences database. [file 12889_2021_11790_MOESM1_ESM.docx]

**Search strategies used in Medline, Embase and Global Health, CINAHL and Web of Sciences databases**

**Ovid MEDLINE(R) and Embase and Global health and Epub Ahead of Print, In-Process & Other Non-Indexed Citations and Daily 2010 to May 2020**

**The following search strategy was applied in Medline, Embase and Global Health**

Tools

1. technolog*.ab,ti.

2. software/ or exp mobile applications/

3. “mobile application*” .ab,ti

4. software.ab,ti.

5. electronic*.ab,ti.

6. automate*.ab,ti.

7. informatic*.ab,ti.

8. dashboard*.ab,ti.

9. “public health informatic*”.ab,ti.

10. device*.ab,ti.

11. tablet*.ab,ti.

12. mobile.ab,ti.

13. mhealth.ab,ti.

14. “mobile health”.ab,ti.

15. “information system*”.ab,ti.

16. “smart phone*”.ab,ti

17. smartphone*.ab,ti.

18. mobile phone/

19. “mobile phone*” .ab,ti

20. toolki*.ab,ti.

22. “situation report*”.ab,ti.

22. sitrep*.ab,ti.

23. (program or programs).ab,ti.

24. 1 or 2 or 3 or 4 or 5 or 6 or 7 or 8 or 9 or 10 or 11 or 12 or 13 or 14 or 15 or 16 or 17 or 18 or 19 or 20 or 21 or 22 or 23

Data collection, management and analysis

25. “data interpretation*”.ab,ti.

26. “descriptive statistic*”.ab,ti.

27. “data visuali#ation”.ab,ti.

28. dataviz.ab,ti.

29. infographic*.ab,ti.

30. “data base management”.ab,ti.

31. “data handling”.ab,ti.

32. “data processing”.ab,ti.

33. “information management”.ab,ti.

34. “database management”.ab,ti.

35. “data organi#ation”.ab,ti.

36. “data aggregation”.ab,ti.

37. “database quality”.ab,ti.

38. “data collection method*”.ab,ti.

39. georeferenc*.ab,ti.

40. mapping.ab,ti.

41. GIS.ab,ti.

42. “geographic information system*”.ab,ti.

43. “data acquisition”.ab,ti.

44. “data compilation”.ab,ti.

45. data collection/

| 46. exp data interpretation, statistical/ or exp spatial analysis/  47. “spatial analys*” .ab,ti  48. (data adj3 management).ab,ti.  49. (data adj3 collection).ab,ti.  50. (data adj3 analysis).ab,ti.  51. (data adj3 capture).ab,ti.  52. exp information processing/  53. “information processing” .ab,ti  54. 25 or 26 or 27 or 28 or 29 or 30 or 31 or 32 or 33 or 34 or 35 or 36 or 37 or 38 or 39 or 40 or 41 or 42 or 43 or 44 or 45 or 46 or 47 or 48 or 49 or 50 or 51 or 52 or 53  Outbreak  55. “disease outbreak*”.ab,ti.  56. exp Disease Outbreaks/  57. “communicable disease*”.ab,ti.  58. epidemic*.ab,ti.  59. “public health surveillance”.ab,ti.  60. exp Public Health Surveillance/  61.”disease detection”.ab,ti.  62. “public health emergenc*”.ab,ti.  63. “disease surveillance”.ab,ti.  64. disease notification/ or epidemiological monitoring/  65. “disease notification” .ab,ti  66. “epidemiological monitoring” .ab,ti  67. ("ewarn" or "ewars" or "ewar" or "early warning alert and response" or "natural disaster*" or "humanitarian emergenc*" or "humanitarian cris*").ab,ti.  68. 55 or 56 or 57 or 58 or 59 or 60 or 61 or 62 or 63 or 64 or 65 or 66 or 67 |  |
| --- | --- |

LMICs

| \|  \| \| --- \| \| 69. low income country/ \| \| 70. middle income country/ \| \| 71. ((developing or less* developed or under developed or underdeveloped or middle income or low* income or underserved or under served or deprived or poor*) adj (economy or economies)).ti,ab. \| \| 72. ((developing or less* developed or under developed or underdeveloped or middle income or low* income or underserved or under served or deprived or poor*) adj (countr* or nation? or population? or world)).ti,ab. \| \| 73. (low* adj (gdp or gnp or gross domestic or gross national)).ti,ab. \| \| 74. (low adj3 middle adj3 countr*).ti,ab. \| \| 75. (lmic or lmics or third world or lami countr*).ti,ab. \| \| 76. transitional countr*.ti,ab. \| \| 77. global south.ti,ab. \| \| 78. "Africa south of the Sahara"/ \| \| 79. ("africa south of the sahara" or sub-saharan africa or central africa or eastern africa or southern africa or western africa).ti,ab. \| \| 80. Botswana/ \| \| 81. (Botswana or Bechuanaland or Kalahari).ti,ab. \| \| 82. Equatorial Guinea/ \| \| 83. (Equatorial Guinea or Spanish Guinea).ti,ab. \| \| 84. Gabon/ \| \| 85. (Gabon or Gabonese Republic).ti,ab. \| \| 86. Mauritius/ \| \| 87. (Mauritius or Agalega Islands).ti,ab. \| \| 88. Namibia/ \| \| 89. Namibia.ti,ab. \| \| 90. South Africa/ \| \| 91. South Africa.ti,ab. \| \| 92. Angola/ \| \| 93. angola.ti,ab. \| \| 94. Cameroon/ \| \| 95. Cameroon.ti,ab. \| \| 96. Cape Verde/ \| \| 97. (Cape Verde or Cabo Verde).ti,ab. \| \| 98. Congo/ \| \| 99. (congo not ((democratic republic adj3 congo) or congo red or crimean-congo)).ti,ab. \| \| 100. Cote d'Ivoire/ \| \| 101. (Cote d'Ivoire or Ivory Coast).ti,ab. \| \| 102. Ghana/ \| \| 103. (Ghana or Gold Coast).ti,ab. \| \| 104. Kenya/ \| \| 105. kenya.mp. \| \| 106. Lesotho/ \| \| 107. (Lesotho or Basutoland).ti,ab. \| \| 108. Mauritania/ \| \| 109. Mauritania.ti,ab. \| \| 110. Nigeria/ \| \| 111. Nigeria.ti,ab. \| \| 112. "Sao Tome and Principe"/ \| \| 113. (sao tome adj2 principe).ti,ab. \| \| 114. Sudan/ \| \| 115. (Sudan not south sudan).ti,ab. \| \| 116. Swaziland/ \| \| 117. Swaziland.ti,ab. \| \| 118. Zambia/ \| \| 119. (Zambia or Northern Rhodesia).ti,ab. \| \| 120. Benin/ \| \| 121. (Benin or Dahomey).ti,ab. \| \| 122. Burkina Faso/ \| \| 123. (Burkina Faso or Burkina Fasso or Upper Volta).ti,ab. \| \| 124. Burundi/ \| \| 125. Burundi.ti,ab. \| \| 126. Central African Republic/ \| \| 127. (Central African Republic or Ubangi-Shari).ti,ab. \| \| 128. Chad/ \| \| 129. Chad.ti,ab. \| \| 130. Comoros/ \| \| 131. (Comoros or Comoro Islands or Mayotte or Iles Comores).ti,ab. \| \| 132. "Democratic Republic Congo"/ \| \| 133. ((democratic republic adj2 congo) or belgian congo or zaire).ti,ab. \| \| 134. Eritrea/ \| \| 135. Eritrea.ti,ab. \| \| 136. Ethiopia/ \| \| 137. Ethiopia.ti,ab. \| \| 138. Gambia/ \| \| 139. Gambia.ti,ab. \| \| 140. Guinea/ \| \| 141. (Guinea not (New Guinea or Guinea Pig* or Guinea Fowl)).ti,ab. \| \| 142. Guinea-Bissau/ \| \| 143. (Guinea-Bissau or Portuguese Guinea).ti,ab. \| \| 144. Liberia/ \| \| 145. Liberia.ti,ab. \| \| 146. Madagascar/ \| \| 147. (Madagascar or Malagasy Republic).ti,ab. \| \| 148. Malawi/ \| \| 149. (Malawi or Nyasaland).ti,ab. \| \| 150. Mali/ \| \| 151. Mali.ti,ab. \| \| 152. Mozambique/ \| \| 153. (Mozambique or Mocambique or Portuguese East Africa).ti,ab. \| \| 154. Niger/ \| \| 155. (Niger not (Aspergillus or Peptococcus or Schizothorax or Cruciferae or Gobius or Lasius or Agelastes or Melanosuchus or radish or Parastromateus or Orius or Apergillus or Parastromateus or Stomoxys)).ti,ab. \| \| 156. Rwanda/ \| \| 157. (Rwanda or Ruanda).ti,ab. \| \| 158. Senegal/ \| \| 159. senegal.ti,ab. \| \| 160. Sierra Leone/ \| \| 161. Sierra Leone.mp. \| \| 162. exp Somalia/ \| \| 163. Somalia.ti,ab. \| \| 164. South Sudan/ \| \| 165. south sudan.ti,ab. \| \| 166. Tanzania/ \| \| 167. (Tanzania or Tanganyika or Zanzibar).ti,ab. \| \| 168. Togo/ \| \| 169. (Togo or Togolese Republic).ti,ab. \| \| 170. Uganda/ \| \| 171. Uganda.ti,ab. \| \| 172. Zimbabwe/ \| \| 173. (Zimbabwe or Rhodesia).ti,ab. \| \| 174. Maldives/ \| \| 175. Maldives.ti,ab. \| \| 176. Algeria/ \| \| 177. Algeria.ti,ab. \| \| 178. Iran/ \| \| 179. Iran.ti,ab. \| \| 180. exp Iraq/ \| \| 181. Iraq.ti,ab. \| \| 182. Jordan/ \| \| 183. Jordan.ti,ab. \| \| 184. Lebanon/ \| \| 185. Lebanon.ti,ab. \| \| 186. Libyan Arab Jamahiriya/ \| \| 187. Libya.ti,ab. \| \| 188. Argentina/ \| \| 189. Argentina.ti,ab. \| \| 190. Belize/ \| \| 191. Belize.ti,ab. \| \| 192. exp Brazil/ \| \| 193. Brazil.ti,ab. \| \| 194. Colombia/ \| \| 195. Colombia.ti,ab. \| \| 196. Costa Rica/ \| \| 197. Costa Rica.ti,ab. \| \| 198. Cuba/ \| \| 199. Cuba.ti,ab. \| \| 200. Dominica/ \| \| 201. Dominica.ti,ab. \| \| 202. Dominican Republic/ \| \| 203. Dominican Republic.ti,ab. \| \| 204. Ecuador/ \| \| 205. Ecuador.ti,ab. \| \| 206. Grenada/ \| \| 207. Grenada.ti,ab. \| \| 208. Guyana/ \| \| 209. Guyana.mp. \| \| 210. Jamaica/ \| \| 211. Jamaica.ti,ab. \| \| 212. Mexico/ \| \| 213. Mexico.ti,ab. \| \| 214. exp Panama/ \| \| 215. Panama.ti,ab. \| \| 216. Paraguay/ \| \| 217. Paraguay.mp. \| \| 218. Peru/ \| \| 219. Peru.ti,ab. \| \| 220. Saint Lucia/ \| \| 221. (St Lucia or Saint Lucia).ti,ab. \| \| 222. "Saint Vincent and the Grenadines"/ \| \| 223. Grenadines.ti,ab. \| \| 224. Suriname/ \| \| 225. Suriname.ti,ab. \| \| 226. Venezuela/ \| \| 227. Venezuela.ti,ab. \| \| 228. Albania/ \| \| 229. Albania.ti,ab. \| \| 230. Azerbaijan/ \| \| 231. Azerbaijan.ti,ab. \| \| 232. Belarus/ \| \| 233. (belarus or byelarus or belorussia).ti,ab. \| \| 234. exp "Bosnia and Herzegovina"/ \| \| 235. (bosnia or herzegovina).ti,ab. \| \| 236. Bulgaria/ \| \| 237. Bulgaria.ti,ab. \| \| 238. Croatia/ \| \| 239. croatia.ti,ab. \| \| 240. Kazakhstan/ \| \| 241. (Kazakhstan or kazakh).ti,ab. \| \| 242. "Macedonia (Republic)"/ \| \| 243. Macedonia.ti,ab. \| \| 244. "Montenegro (republic)"/ \| \| 245. Montenegro.ti,ab. \| \| 246. Romania/ \| \| 247. Romania.ti,ab. \| \| 248. exp Russian Federation/ \| \| 249. USSR/ \| \| 250. (Russia or Russian Federation or USSR or Union Soviet Socialist Republics or Soviet Union).mp. \| \| 251. exp Serbia/ \| \| 252. serbia.ti,ab. \| \| 253. "Turkey (republic)"/ \| \| 254. turkey.ti,ab. not animal/ \| \| 255. Turkmenistan/ \| \| 256. Turkmenistan.ti,ab. \| \| 257. Yugoslavia/ \| \| 258. yugoslavia.ti,ab. \| \| 259. exp Samoan Islands/ \| \| 260. american samoa.ti,ab. \| \| 261. exp China/ \| \| 262. china.ti,ab. \| \| 263. Fiji/ \| \| 264. fiji.ti,ab. \| \| 265. Malaysia/ \| \| 266. malaysia.ti,ab. \| \| 267. Marshall Islands/ \| \| 268. marshall islands.ti,ab. \| \| 269. Nauru/ \| \| 270. nauru.ti,ab. \| \| 271. ("independent state of samoa" or (samoa not american samoa) or western samoa or navigator islands or samoan islands).ti,ab. \| \| 272. Thailand/ \| \| 273. Thailand.ti,ab. \| \| 274. Tonga/ \| \| 275. tonga.ti,ab. \| \| 276. Tuvalu/ \| \| 277. Tuvalu.ti,ab. \| \| 278. Bangladesh/ \| \| 279. Bangladesh.ti,ab. \| \| 280. Bhutan/ \| \| 281. Bhutan.ti,ab. \| \| 282. exp India/ \| \| 283. India.ti,ab. \| \| 284. exp Pakistan/ \| \| 285. Pakistan.ti,ab. \| \| 286. Sri Lanka/ \| \| 287. Sri Lanka.ti,ab. \| \| 288. Djibouti/ \| \| 289. (Djibouti or French Somaliland).ti,ab. \| \| 290. Egypt/ \| \| 291. Egypt.ti,ab. \| \| 292. Jordan/ \| \| 293. Jordan.ti,ab. \| \| 294. Morocco/ \| \| 295. Morocco.ti,ab. \| \| 296. Syrian Arab Republic/ \| \| 297. (Syria or Syrian Arab Republic).ti,ab. \| \| 298. Tunisia/ \| \| 299. tunisia.mp. \| \| 300. Palestine/ \| \| 301. Gaza.ti,ab. \| \| 302. Yemen/ \| \| 303. Yemen.ti,ab. \| \| 304. Bolivia/ \| \| 305. Bolivia.ti,ab. \| \| 306. El Salvador/ \| \| 307. El Salvador.ti,ab. \| \| 308. Guatemala/ \| \| 309. Guatemala.ti,ab. \| \| 310. Honduras/ \| \| 311. Honduras.ti,ab. \| \| 312. Nicaragua/ \| \| 313. Nicaragua.ti,ab. \| \| 314. Armenia/ \| \| 315. Armenia.ti,ab. \| \| 316. "Georgia (Republic)"/ \| \| 317. Kosovo/ \| \| 318. Kosovo.ti,ab. \| \| 319. Kyrgyzstan/ \| \| 320. (kyrgyzstan or kyrgyz republic or kirghizia or kirghiz).ti,ab. \| \| 321. Moldova/ \| \| 322. Moldova.ti,ab. \| \| 323. Tajikistan/ \| \| 324. tajikistan.ti,ab. \| \| 325. exp Ukraine/ \| \| 326. Ukraine.ti,ab. \| \| 327. Uzbekistan/ \| \| 328. Uzbekistan.ti,ab. \| \| 329. Cambodia/ \| \| 330. cambodia.ti,ab. \| \| 331. exp Indonesia/ \| \| 332. indonesia.ti,ab. \| \| 333. Kiribati/ \| \| 334. Kiribati.ti,ab. \| \| 335. Laos/ \| \| 336. (laos or (lao adj1 democratic republic)).ti,ab. \| \| 337. "Marshall Islands"/ \| \| 338. "Federated States of Micronesia"/ \| \| 339. (marshall island* or caroline island* or ellice island* or gilbert island* or johnston island* or marianaisland* or micronesia or pacific island*).ti,ab \| \| 340. Mongolia/ \| \| 341. mongolia.ti,ab. \| \| 342. Myanmar/ \| \| 343. (myanmar or burma).ti,ab. \| \| 344. Papua New Guinea/ \| \| 345. Papua New Guinea.ti,ab. \| \| 346. Philippines/ \| \| 347. Philippines.ti,ab. \| \| 348. Timor-Leste/ \| \| 349. Timor-Leste.ti,ab. \| \| 350. Vanuatu/ \| \| 351. Vanuatu.ti,ab. \| \| 352. Viet Nam/ \| \| 353. (Viet Nam or vietnam).ti,ab. \| \| 354. Afghanistan/ \| \| 355. Afghanistan.ti,ab. \| \| 356. Nepal/ \| \| 357. Nepal.ti,ab. \| \| 358. Haiti/ \| \| 359. Haiti.ti,ab. \| \| 360. "North Korea"/ \| \| 361. (north korea or (democratic people* republic adj2 korea)).ti,ab.  362. developing country/ \| \| 363. or/69-362 \| \| 364. 24 and 54 and 68 and 363 \| \| 365. limit 364 to ((english or french or german or portuguese or spanish) and yr="2010 -Current") \| |  |
| --- | --- | --- | --- | --- | --- | --- | --- | --- | --- | --- | --- | --- | --- | --- | --- | --- | --- | --- | --- | --- | --- | --- | --- | --- | --- | --- | --- | --- | --- | --- | --- | --- | --- | --- | --- | --- | --- | --- | --- | --- | --- | --- | --- | --- | --- | --- | --- | --- | --- | --- | --- | --- | --- | --- | --- | --- | --- | --- | --- | --- | --- | --- | --- | --- | --- | --- | --- | --- | --- | --- | --- | --- | --- | --- | --- | --- | --- | --- | --- | --- | --- | --- | --- | --- | --- | --- | --- | --- | --- | --- | --- | --- | --- | --- | --- | --- | --- | --- | --- | --- | --- | --- | --- | --- | --- | --- | --- | --- | --- | --- | --- | --- | --- | --- | --- | --- | --- | --- | --- | --- | --- | --- | --- | --- | --- | --- | --- | --- | --- | --- | --- | --- | --- | --- | --- | --- | --- | --- | --- | --- | --- | --- | --- | --- | --- | --- | --- | --- | --- | --- | --- | --- | --- | --- | --- | --- | --- | --- | --- | --- | --- | --- | --- | --- | --- | --- | --- | --- | --- | --- | --- | --- | --- | --- | --- | --- | --- | --- | --- | --- | --- | --- | --- | --- | --- | --- | --- | --- | --- | --- | --- | --- | --- | --- | --- | --- | --- | --- | --- | --- | --- | --- | --- | --- | --- | --- | --- | --- | --- | --- | --- | --- | --- | --- | --- | --- | --- | --- | --- | --- | --- | --- | --- | --- | --- | --- | --- | --- | --- | --- | --- | --- | --- | --- | --- | --- | --- | --- | --- | --- | --- | --- | --- | --- | --- | --- | --- | --- | --- | --- | --- | --- | --- | --- | --- | --- | --- | --- | --- | --- | --- | --- | --- | --- | --- | --- | --- | --- | --- | --- | --- | --- | --- | --- | --- | --- | --- | --- | --- | --- | --- | --- | --- | --- | --- | --- | --- | --- | --- | --- | --- | --- | --- | --- | --- | --- | --- | --- |
|  |  |
|  |  |

**CINAHL (limit to 2010-Current and** english or french or german or portuguese or Spanish)

| S1 | technolog* or software or "mobile application*" or electronic* or automate* or informatic* or dashboard* or  "public health informatic*" or device* or tablet* or mobile or mhealth or "mobile health" or information* or  "smart phone*" or smartphones* or "mobile phone*" or toolki* or "situation report*" or sitrep* or program or  programs |
| --- | --- |
| S2 | "data interpretation*" or "descriptive statistic*" or "data visuali#ation" or dataviz or infographic* or  "data base management" or "data handling" or "data processing" or "information management" or  "database management" or "data organi#ation" or "data aggregation" or "database quality" or "data collection  method*" or georeferenc* or mapping or GIS or "geographic information system*" or "data acquisition" or  "data compilation" or "data collection" or "data interpretation" or "spatial analys*" or "data management" or  "data analysis" or "data capture" or "information processing" |
| S3 | "disease outbreak*" or "communicable disease*" or epidemic* or "public health surveillance" or  "disease detection" or "public health emergenc*" or "disease surveillance" or "disease notification" or  "epidemiological monitoring" or "ewarn" or "ewars" or "ewar" or "early warning alert and response" or  "natural disaster*" or "humanitarian emergenc*" or "humanitarian cris*" |
| S4 | "developing country" or "low income country" or "middle income country" or "developing econom*" or  "less developed econom*" or "underdeveloped econom*" or "middle income econom*" or  "low income econom*" or "underserved econom*" or "deprived econom*" or "poor econom*" or  "developing countr*" or "less developed countr*" or "underdeveloped countr*" or "middle income countr*" or  "low income countr*" or "underserved countr*" or "deprived countr*" or "poor countr*" or "developing nation*"  or "less developed nation*" or "underdeveloped nation*" or "middle income nation*" or "low income nation*"  or "underserved nation*" or "deprived nation*" or "poor nation*" or "developing world" or  "less developed world" or "underdeveloped world" or "middle income world" or "low income world" or  "underserved world" or "deprived world" or "poor world" or "low gdp" or "low gnp" or "low gross domestic"  or "low gross national" or "lmic*" or "third world" or "lami countr*" or "transitional countr*" or "global south"  or "africa south of the sahara" or "sub-saharan africa" or "central africa" or "eastern africa" or  "southern africa" or "western africa" or botswana or bechuanaland or kalahari or "equatorial guinea" or  "spanish guinea" or gabon or "gabonese republic" or mauritius or "agalega islands" or namibia or  "south africa" or angola or cameroon or "cape verde" or "cabo verde" or congo or "cote d'ivoire" or  "ivory coast" or ghana or "gold coast" or kenya or lesotho or basutoland or mauritania or nigeria or  "sao tome and prinicipe" or sudan or "south sudan" or swaziland or zambia or "northern rhodesia" or  benin or dahmoey or "burkina faso" or "burkina fasso" or "upper volta" or burundi or "central african republic"  or "ubangi-shari" or chad or comores or "comoro islands " or mayotte or "iles comores" or  "democratic republic congo" or "belgian congo" or zaire or eritrea or ethiopia or gambia or guinea or  guinea-bissau or "portuguese guinea" or liberia or madagascar or "malagasy republic"or malawi or  nyasaland or mali or mozambique or mocambique or "portuguese east africa" or niger or rwanda or  ruanda or senegal or "sierra leone" or somalia or tanzania or tanganyika or zanzibar or togo or  "togolese republic" or uganda or zimbabwe or rhodesia or maldives or algeria or iran or iraq or  jordan or lebanon or "libyan arab jamahiriya" or libya or argentina or belize or brazil or colombia or  "costa rica" or cuba or dominica or "dominican republic" or ecuador or grenada or guyana or jamaica  or mexico or panama or paraguay or peru or "saint lucia" or "st lucia" or "saint vincent and the grenadines"  or grenadines or suriname or venezuela or albania or azerbaijan or belarus or byelarus or belorussia or  "bosnia and herzegovina" or bosnia or herzegovina or bulgaria or croatia or kazakhstan or kazakh or  macedonia or montenegro or romania or "russian federation" or USSR or russia or  "union soviet socialist republics" or "soviet union" or serbia or turkey or turkmenistan or  yugoslavia or "samoan islands" or "american samoa" or china or fiji or malaysia or "marshall island*" or  naura or "independent state of samoa" or "western samoa" or "navigator islands" or "samoan islands" or  thailand or tonga or tuvalu or bangladesh or bhutan or india or pakistan or "sri lanka" or djibouti or  "french somaliland" or egypt or jordan or morocco or "syrian arab republic" or syria or tunisia or  palestine or gaza or yemen or bolivia or "el salvador" or guatemala or honduras or nicaragua or  armenia or georgia or kosovo or kyrgyzstan or "kyrgyz republic" or kirghizia or kighiz or moldova or  tajikistan or ukraine or uzbekistan or cambodia or indonesia or kiribati or laos or  "laos democratic republic" or "federated states of micronesia" or "caroline island*"  or "ellice island*" or "gilbert island*" or "johnston island*" or marianaisland* or  micronesia or "pacific island*" or mongolia or myanmar or burma or "papua new guinea"  or philippines or timor-leste or vanuatu or "viet nam" or vietnam or afghanistan or nepal or  haiti or "north korea" or "democratic people* republic of korea" |
| S5 | S1 AND S2 AND S3 AND S4 |

**Web of Science, 2010- 2020, French, Spanish, Portuguese, German or English**

1. TS = (technolog* or software or mobile applications or electronic* or automate* or informatic* or dashboard* or public health informatic* or device* or tablet* or mobile or mhealth or mobile health or information* or smart phone* or smartphones* or mobile phone* or toolki* or situation report* or sitrep* or program or programs)
2. TS= ("data interpretation*" or "descriptive statistic*" or "data visuali#ation" or dataviz or infographic* or "data base management" or "data handling" or "data processing" or "information management" or "database management" or "data organi#ation" or "data aggregation" or "database quality" or "data collection method*" or georeferenc* or mapping or GIS or "geographic information system*" or "data acquisition" or "data compilation" or "data collection" or "data interpretation" or "spatial analys*" or "data management" or "data analysis" or "data capture" or "information processing")
3. TS = (developing country or low income country or middle income country or developing econom* or less developed econom* or underdeveloped econom* or middle income econom* or low income econom* or underserved econom* or deprived econom* or poor econom* or developing countr* or less developed countr* or underdeveloped countr* or middle income countr* or low income countr* or underserved countr* or deprived countr* or poor countr* or developing nation* or less developed nation* or underdeveloped nation* or middle income nation* or low income nation* or underserved nation* or deprived nation* or poor nation* or developing world or less developed world or underdeveloped world or middle income world or low income world or underserved world or deprived world or poor world or low gdp or low gnp or low gross domestic or low gross national or lmic* or third world or lami countr* or transitional countr* or global south or ("africa south of the sahara" or sub-saharan africa or central africa or eastern africa or southern africa or western africa or botswana or bechuanaland or kalahari or equatorial guinea or spanish guinea or gabon or gabonese republic or mauritius or agalega islands or namibia or south africa or angola or cameroon or cape verde or cabo verde or congo or cote d'ivoire or ivory coast or ghana or gold coast or kenya or lesotho or basutoland or mauritania or nigeria or sao tome and prinicipe or sudan or south sudan or swaziland or zambia or northern rhodesia or benin or dahmoey or burkina faso or burkina fasso or upper volta or burundi or central african republic or ubangi-shari or chad or comores or comoro islands or mayotte or iles comores or democratic republic congo or belgian congo or zaire or eritrea or ethiopia or gambia or guinea or guinea-bissau or portuguese guinea or liberia or madagascar or malagasy republic or malawi or nyasaland or mali or mozambique or mocambique or portuguese east africa or niger or rwanda or ruanda or senegal or sierra leone or somalia or tanzania or tanganyika or zanzibar or togo or togolese republic or uganda or zimbabwe or rhodesia or maldives or algeria or iran or iraq or jordan or lebanon or libyan arab jamahiriya or libya or argentina or belize or brazil or colombia or costa rica or cuba or dominica or dominican republic or ecuador or grenada or guyana or jamaica or mexico or panama or paraguay or peru or saint lucia or st lucia or saint vincent and the grenadines or grenadines or suriname or venezuela or albania or azerbaijan or belarus or byelarus or belorussia or bosnia and herzegovina or bosnia or herzegovina or bulgaria or croatia or kazakhstan or kazakh or macedonia or montenegro or romania or russian federation or USSR or russia or union soviet socialist republics or soviet union or serbia or turkey or turkmenistan or yugoslavia or samoan islands or american samoa or china or fiji or malaysia or marshall islands or naura or independent state of samoa or western samoa or navigator islands or samoan islands or thailand or tonga or tuvalu or bangladesh or bhutan or india or pakistan or sri lanka or djibouti or french somaliland or egypt or jordan or morocco or syrian arab republic or syria or tunisia or palestine or gaza or yemen or bolivia or el salvador or guatemala or honduras or nicaragua or armenia or georgia or kosovo or kyrgyzstan or kyrgyz republic or kirghizia or kighiz or moldova or tajikistan or ukraine or uzbekistan or cambodia or indonesia or kiribati or laos or laos democratic republic or marshall island* or federated states of micronesia or caroline island* or ellice island* or gilbert island* or johnston island* or marianaisland* or micronesia or pacific island* or mongolia or myanmar or burma or papua new guinea or philippines or timor-leste or vanuatu or viet nam or vietnam or afghanistan or nepal or haiti or north korea or democratic people* republic of korea)
4. TS = (“disease outbreak*” or ”communicable disease*” or epidemic* or “public health surveillance“or “disease detection” or “public health emergenc*” or “disease surveillance” or “disease notification” or “epidemiological monitoring” or "ewarn" OR "ewars" OR "early warning alert and response" or "ewar" or "humanitarian cris*" or "humanitarian emergenc*" or "natural disaster*")

1 and 2 and 3 and 4
