# Supplementary material for: Electronic data collection, management and analysis tools used for outbreak response in low- and middle-income countries: a systematic review and stakeholder survey
Source: BMC Public Health. 2021 Sep 25;21:1741. doi: 10.1186/s12889-021-11790-w (PMC8464108; doi:10.1186/s12889-021-11790-w)
Supplement: Supplementary file 2 — Additional file 2. Definitions used for data extraction. Table shows terms used for technical characteristics of electronic tools examined and their definitions. [file 12889_2021_11790_MOESM2_ESM.docx]

**Definitions used for data extraction**

| **Term** | **Definition** |
| --- | --- |
| **Data encryption** | a data security measure allowing the encoding of data into another format that requires a key/password to decrypt |
| **Data management** | processes allowing collection, validation and storage of data |
| **Data storage** | location where data collected by an electronic tool are stored i.e. on a local server and/or on the cloud |
| **Data visualisation** | a functionality of an electronic tool that allows creation of graphical representations (charts, plots etc) of data collected |
| **Free services** | refers to whether or not all functionalities of the tool are available at no cost |
| **Free software** | refers to whether or not the electronic tool is freely accessible e.g. available on Google Play Store at no cost |
| **License type** | whether or not the tool is open source/semi-open/freeware or proprietary |
| **Offline data collection** | refers to the capacity of an electronic tool to collect and store data without an internet connection and to subsequently send data once an internet connection becomes available |
| **Operating system compatibility** | refers to the ability of electronic tools to function on specific operating systems |
| **Web data entry** | a functionality of an electronic tool permitting collection of data on desktops or mobile devices using an internet browser rather than a mobile application |
