# Supplementary material for: Electronic data collection, management and analysis tools used for outbreak response in low- and middle-income countries: a systematic review and stakeholder survey
Source: BMC Public Health. 2021 Sep 25;21:1741. doi: 10.1186/s12889-021-11790-w (PMC8464108; doi:10.1186/s12889-021-11790-w)
Supplement: Supplementary file 3 — Additional file 3. Data dictionary of the online Enketo Stakeholder survey on electronic data collection, management and analysis tools. The data dictionary shows the variable names, question text, response options/question type, skip logic and validation information as well as whether a question was required or not. [file 12889_2021_11790_MOESM3_ESM.docx]

Data dictionary of online Enketo Stakeholder survey on electronic data collection, management and analysis tools

| **Variable name** | **Question** | **Skip logic** | **Response options** | **Acceptable data entry range** | **Guidance on data entry** | **Required question** |
| --- | --- | --- | --- | --- | --- | --- |
| today | Today's date |  | Date/Time |  |  |  |
| more_info | Would you like more information on this study before proceeding to the informed consent? |  | Select one: Yes, No |  |  |  |
| agree_consent | Do you consent to participate in this study? | ${more_info} = 'no' | Select one: Yes, No |  |  |  |
| name | Name |  | Text |  |  | yes |
| email | Email address |  | Text |  |  | yes |
| organisation | Name of your organisation |  | Select one: African CDC, ALIMA, US-CDC, EAN, EMPHNET, Global health network, GOARN, IFRC, ITM, Map Action, Ministry of Health, MSF, OCHA, Red Cross, SAFETYnet, Save the children, TEPHINET, WHO, Other |  |  | yes |
| moh_country | Please specify the country that you are representing | ${organisation} = 'moh' | Select one: Aghanistan, Bangladesh, Cameroon, Central African Republic, Chad, Democratic Republic of Congo, Ethiopia, Iran, Iraq, Mali, Nigeria, Occupied Palestinian Territory, Pakistan, Somalia, South Sudan, Sudan, Syrian Arab Republic, Turkey/North Syria, Ukraine, Yemen, Not applicable |  |  | yes |
| other_org | Please specify the name of your organisation | ${organisation} = 'other' | Text |  |  | yes |
| role | What is your role in the organisation? |  | Select one: Data analyst, Data manager, Data scientist, Epidemiologist, Field coordinator, Other |  |  | yes |
| other_role | Please specify your role | ${role} = 'other' | Text |  |  |  |
| level | At which operational levels do you work within your organisation? |  | Select all that apply: Field, Headquarters, National, Regional, Other |  |  | yes |
| other_level | Please specify the levels that you work within your organisation? | selected(${level},'other') | Text |  |  |  |
| country | Have you responded to outbreaks in any of the following countries? Please tick all that apply |  | Select all that apply: Aghanistan, Bangladesh, Cameroon, Central African Republic, Chad, Democratic Republic of Congo, Ethiopia, Iran, Iraq, Mali, Nigeria, Occupied Palestinian Territory, Pakistan, Somalia, South Sudan, Sudan, Syrian Arab Republic, Turkey/North Syria, Ukraine, Yemen, Not applicable | not(selected(.,  'na') and count-selected(.) >=2) | You cannot choose **not applicable** and another country for this answer. | yes |
| tool | Have you been involved in any of the following activities in outbreak response in any of the previously highlighted countries? Please tick all that apply |  | Select all that apply: Data analysis, Data collection, Data management, Development of data collection forms, Not applicable | not(selected(.,  'na') and count-selected(.) >=2) | You cannot choose **not applicable** and other activities for this answer. | yes |
| coll_tool | Which electronic **DATA COLLECTION** tool(s) have you used when responding to outbreaks? |  | Select all that apply: ARGUS, AVADAR, CommCare, Dharma, DHIS2, eDEWS, EpiCollect, EpiInfo, eNIHS, EWARS, Go.data, Kobo Toolbox, Magpi, Open Data Kit (ODK), OpenMRS, RapidPro, REDCap, SORMAS, SurveyCTO, Other |  |  | yes |
| other_collect_name | Please specify the name of the data collection tool | selected(${coll_tool}, 'other') | Text |  |  |  |
| alerts | Alert management |  | Select one: Yes, No |  |  | yes |
| case_investigation | Case investigation |  | Select one: Yes, No |  |  | yes |
| contact_tracing | Contact tracing |  | Select one: Yes, No |  |  | yes |
| health_promotion | To collect health promotion data |  | Select one: Yes, No |  |  | yes |
| hospital | To collect clinical data |  | Select one: Yes, No |  |  | yes |
| ipc | To conduct IPC assessments |  | Select one: Yes, No |  |  | yes |
| lab | To collect laboratory data |  | Select one: Yes, No |  |  | yes |
| surveillance | To collect surveillance data |  | Select one: Yes, No |  |  | yes |
| trials | To conduct clinical trials |  | Select one: Yes, No |  |  | yes |
| wash | To conduct WASH assessments |  | Select one: Yes, No |  |  | yes |
| other_argus | Other |  | Select one: Yes, No |  |  | yes |
| other_argus_purpose | Please specify the other use of **ARGUS** | ${other_argus} = 'yes' | Text |  |  |  |
| rate_coll_argus | How would you rate the usefulness of **ARGUS**? |  | Select one: 1- not useful, 2, 3, 4, 5- very useful |  |  | yes |
| ease_coll_argus | How would you rate the ease of use of **ARGUS**? |  | Select one: 1- difficult to use, 2, 3, 4, 5- easy to use |  |  | yes |
| improvement_coll_argus | How could **ARGUS** be improved, if at all? |  | Text |  |  | yes |
| reason_coll_argus | Why was **ARGUS** chosen over other available tools? |  | Text |  |  | yes |
| users_coll_argus | Who are the main users of ARGUS in your organisation? |  | Select all that apply: Data analyst, Data manager, Data scientist, Epidemiologist, Field coordinator, Field data collectors, Other |  |  | yes |
| users_coll_argus_other | Please specify the other users of **ARGUS** | selected(${users_coll_argus}, 'other') | Text |  |  |  |
| alerts | Alert management |  | Select one: Yes, No |  |  | yes |
| case_investigation | Case investigation |  | Select one: Yes, No |  |  | yes |
| contact_tracing | Contact tracing |  | Select one: Yes, No |  |  | yes |
| health_promotion | To collect health promotion data |  | Select one: Yes, No |  |  | yes |
| hospital | To collect clinical data |  | Select one: Yes, No |  |  | yes |
| ipc | To conduct IPC assessments |  | Select one: Yes, No |  |  | yes |
| lab | To collect laboratory data |  | Select one: Yes, No |  |  | yes |
| surveillance | To collect surveillance data |  | Select one: Yes, No |  |  | yes |
| trials | To conduct clinical trials |  | Select one: Yes, No |  |  | yes |
| wash | To conduct WASH assessments |  | Select one: Yes, No |  |  | yes |
| other_avadar | Other |  | Select one: Yes, No |  |  | yes |
| other_avadar_purpose | Please specify other use(s) of **AVADAR** | ${other_avadar} = 'yes' | Text |  |  | yes |
| rate_coll_avadar | How would you rate the usefulness of **AVADAR**? |  | Select one: 1- not useful, 2, 3, 4, 5- very useful |  |  | yes |
| ease_coll_avadar | How would you rate the ease of use of **AVADAR**? |  | Select one: 1- difficult to use, 2, 3, 4, 5- easy to use |  |  | yes |
| improvement_coll_avadar | How could **AVADAR** be improved, if at all? |  | Text |  |  | yes |
| reason_coll_avadar | Why was **AVADAR** chosen over other available tools? |  | Text |  |  | yes |
| users_coll_avadar | Who are the main users of **AVADAR** in your organisation? |  | Select all that apply: Data analyst, Data manager, Data scientist, Epidemiologist, Field coordinator, Field data collectors, Other |  |  | yes |
| users_coll_avadar_other | Please specify the other users of **AVADAR** | selected(${users_coll_avadar}, 'other') | Text |  |  |  |
| alerts | Alert management |  | Select one: Yes, No |  |  | yes |
| case_investigation | Case investigation |  | Select one: Yes, No |  |  | yes |
| contact_tracing | Contact tracing |  | Select one: Yes, No |  |  | yes |
| health_promotion | To collect health promotion data |  | Select one: Yes, No |  |  | yes |
| hospital | To collect clinical data |  | Select one: Yes, No |  |  | yes |
| ipc | To conduct IPC assessments |  | Select one: Yes, No |  |  | yes |
| lab | To collect laboratory data |  | Select one: Yes, No |  |  | yes |
| surveillance | To collect surveillance data |  | Select one: Yes, No |  |  | yes |
| trials | To conduct clinical trials |  | Select one: Yes, No |  |  | yes |
| wash | To conduct WASH assessments |  | Select one: Yes, No |  |  | yes |
| other_commcare | Other |  | Select one: Yes, No |  |  | yes |
| other_commcare_purpose | Please specify other use(s) of **CommCare** | ${other_commcare} = 'yes' | Text |  |  |  |
| rate_coll_commcare | How would you rate the usefulness of **CommCare**? |  | Select one: 1- not useful, 2, 3, 4, 5- very useful |  |  | yes |
| ease_coll_commcare | How would you rate the ease of use of **CommCare**? |  | Select one: 1- difficult to use, 2, 3, 4, 5- easy to use |  |  | yes |
| improvement_coll_commcare | How could **CommCare** be improved, if at all? |  | Text |  |  | yes |
| reason_coll_commcare | Why was **CommCare** chosen over other available tools? |  | Text |  |  | yes |
| users_coll_commcare | Who are the main users of **CommCare** in your organisation? |  | Select all that apply: Data analyst, Data manager, Data scientist, Epidemiologist, Field coordinator, Field data collectors, Other |  |  | yes |
| users_coll_commcare_other | Please specify the other users of **CommCare** | selected(${users_coll_commcare}, 'other') | Text |  |  |  |
| alerts | Alert management |  | Select one: Yes, No |  |  | yes |
| case_investigation | Case investigation |  | Select one: Yes, No |  |  | yes |
| contact_tracing | Contact tracing |  | Select one: Yes, No |  |  | yes |
| health_promotion | To collect health promotion data |  | Select one: Yes, No |  |  | yes |
| hospital | To collect clinical data |  | Select one: Yes, No |  |  | yes |
| ipc | To conduct IPC assessments |  | Select one: Yes, No |  |  | yes |
| lab | To collect laboratory data |  | Select one: Yes, No |  |  | yes |
| surveillance | To collect surveillance data |  | Select one: Yes, No |  |  | yes |
| trials | To conduct clinical trials |  | Select one: Yes, No |  |  | yes |
| wash | To conduct WASH assessments |  | Select one: Yes, No |  |  | yes |
| other_dharma | Other |  | Select one: Yes, No |  |  | yes |
| other_dharma_purpose | Please specify other use(s) of **Dharma** | ${other_dharma} = 'yes' | Text |  |  |  |
| rate_coll_dharma | How would you rate the usefulness of **Dharma**? |  | Select one: 1- not useful, 2, 3, 4, 5- very useful |  |  | yes |
| ease_coll_dharma | How would you rate the ease of use of **Dharma**? |  | Select one: 1- difficult to use, 2, 3, 4, 5- easy to use |  |  | yes |
| improvement_coll_dharma | How could **Dharma** be improved, if at all? |  | Text |  |  | yes |
| reason_coll_dharma | Why was **Dharma** chosen over other available tools? |  | Text |  |  | yes |
| users_coll_dharma | Who are the main users of **Dharma** in your organisation? |  | Select all that apply: Data analyst, Data manager, Data scientist, Epidemiologist, Field coordinator, Field data collectors, Other |  |  | yes |
| users_coll_dharma_other | Please specify the other users of **Dharma** | selected(${users_coll_dharma}, 'other') | Text |  |  |  |
| alerts | Alert management |  | Select one: Yes, No |  |  | yes |
| case_investigation | Case investigation |  | Select one: Yes, No |  |  | yes |
| contact_tracing | Contact tracing |  | Select one: Yes, No |  |  | yes |
| health_promotion | To collect health promotion data |  | Select one: Yes, No |  |  | yes |
| hospital | To collect clinical data |  | Select one: Yes, No |  |  | yes |
| ipc | To conduct IPC assessments |  | Select one: Yes, No |  |  | yes |
| lab | To collect laboratory data |  | Select one: Yes, No |  |  | yes |
| surveillance | To collect surveillance data |  | Select one: Yes, No |  |  | yes |
| trials | To conduct clinical trials |  | Select one: Yes, No |  |  | yes |
| wash | To conduct WASH assessments |  | Select one: Yes, No |  |  | yes |
| other_dhis2 | Other |  | Select one: Yes, No |  |  | yes |
| other_dhis2_purpose | Please specify other use(s) of **DHIS2** | ${other_dhis2} = 'yes' | Text |  |  |  |
| rate_coll_dhis2 | How would you rate the usefulness of **DHIS2**? |  | Select one: 1- not useful, 2, 3, 4, 5- very useful |  |  | yes |
| ease_coll_dhis2 | How would you rate the ease of use of **DHIS2**? |  | Select one: 1- difficult to use, 2, 3, 4, 5- easy to use |  |  | yes |
| improvement_coll_dhis2 | How could **DHIS2** be improved, if at all? |  | Text |  |  | yes |
| reason_coll_dhis2 | Why was **DHIS2** chosen over other available tools? |  | Text |  |  | yes |
| users_coll_dhis2 | Who are the main users of **DHIS2** in your organisation? |  | Select all that apply: Data analyst, Data manager, Data scientist, Epidemiologist, Field coordinator, Field data collectors, Other |  |  | yes |
| users_coll_dhis2_other | Please specify the other users of **DHIS2** | selected(${users_coll_dhis2}, 'other') | Text |  |  |  |
| alerts | Alert management |  | Select one: Yes, No |  |  | yes |
| case_investigation | Case investigation |  | Select one: Yes, No |  |  | yes |
| contact_tracing | Contact tracing |  | Select one: Yes, No |  |  | yes |
| health_promotion | To collect health promotion data |  | Select one: Yes, No |  |  | yes |
| hospital | To collect clinical data |  | Select one: Yes, No |  |  | yes |
| ipc | To conduct IPC assessments |  | Select one: Yes, No |  |  | yes |
| lab | To collect laboratory data |  | Select one: Yes, No |  |  | yes |
| surveillance | To collect surveillance data |  | Select one: Yes, No |  |  | yes |
| trials | To conduct clinical trials |  | Select one: Yes, No |  |  | yes |
| wash | To conduct WASH assessments |  | Select one: Yes, No |  |  | yes |
| other_edews | Other |  | Select one: Yes, No |  |  | yes |
| other_edews_purpose | Please specify other use(s) of **eDEWS** | ${other_edews} = 'yes' | Text |  |  |  |
| rate_coll_edews | How would you rate the usefulness of **eDEWS**? |  | Select one: 1- not useful, 2, 3, 4, 5- very useful |  |  | yes |
| ease_coll_edews | How would you rate the ease of use of **eDEWS**? |  | Select one: 1- difficult to use, 2, 3, 4, 5- easy to use |  |  | yes |
| improvement_coll_edews | How could **eDEWS** be improved, if at all? |  | Text |  |  | yes |
| reason_coll_edews | Why was **eDEWS** chosen over other available tools? |  | Text |  |  | yes |
| users_coll_edews | Who are the main users of **eDEWS** in your organisation? |  | Select all that apply: Data analyst, Data manager, Data scientist, Epidemiologist, Field coordinator, Field data collectors, Other |  |  | yes |
| users_coll_edews_other | Please specify the other users of **eDEWS** | selected(${users_coll_edews}, 'other') | Text |  |  |  |
| alerts | Alert management |  | Select one: Yes, No |  |  | yes |
| case_investigation | Case investigation |  | Select one: Yes, No |  |  | yes |
| contact_tracing | Contact tracing |  | Select one: Yes, No |  |  | yes |
| health_promotion | To collect health promotion data |  | Select one: Yes, No |  |  | yes |
| hospital | To collect clinical data |  | Select one: Yes, No |  |  | yes |
| ipc | To conduct IPC assessments |  | Select one: Yes, No |  |  | yes |
| lab | To collect laboratory data |  | Select one: Yes, No |  |  | yes |
| surveillance | To collect surveillance data |  | Select one: Yes, No |  |  | yes |
| trials | To conduct clinical trials |  | Select one: Yes, No |  |  | yes |
| wash | To conduct WASH assessments |  | Select one: Yes, No |  |  | yes |
| other_epicollect | Other |  | Select one: Yes, No |  |  | yes |
| other_epicollect_purpose | Please specify other use(s) of **EpiCollect** | ${other_epicollect} = 'yes' | Text |  |  |  |
| rate_coll_epicollect | How would you rate the usefulness of **EpiCollect**? |  | Select one: 1- not useful, 2, 3, 4, 5- very useful |  |  | yes |
| ease_coll_epicollect | How would you rate the ease of use of **EpiCollect**? |  | Select one: 1- difficult to use, 2, 3, 4, 5- easy to use |  |  | yes |
| improvement_coll_epicollect | How could **EpiCollect** be improved, if at all? |  | Text |  |  | yes |
| reason_coll_epicollect | Why was **EpiCollect** chosen over other available tools? |  | Text |  |  | yes |
| users_coll_epicollect | Who are the main users of **EpiCollect** in your organisation? |  | Select all that apply: Data analyst, Data manager, Data scientist, Epidemiologist, Field coordinator, Field data collectors, Other |  |  | yes |
| users_coll_epicollect_other | Please specify the other users of **EpiCollect** | selected(${users_coll_epicollect}, 'other') | Text |  |  |  |
| alerts | Alert management |  | Select one: Yes, No |  |  | yes |
| case_investigation | Case investigation |  | Select one: Yes, No |  |  | yes |
| contact_tracing | Contact tracing |  | Select one: Yes, No |  |  | yes |
| health_promotion | To collect health promotion data |  | Select one: Yes, No |  |  | yes |
| hospital | To collect clinical data |  | Select one: Yes, No |  |  | yes |
| ipc | To conduct IPC assessments |  | Select one: Yes, No |  |  | yes |
| lab | To collect laboratory data |  | Select one: Yes, No |  |  | yes |
| surveillance | To collect surveillance data |  | Select one: Yes, No |  |  | yes |
| trials | To conduct clinical trials |  | Select one: Yes, No |  |  | yes |
| wash | To conduct WASH assessments |  | Select one: Yes, No |  |  | yes |
| other_epiinfo | Other |  | Select one: Yes, No |  |  | yes |
| other_epiinfo_purpose | Please specify other use(s) of **EpiInfo** | ${other_epiinfo} = 'yes' | Text |  |  |  |
| rate_coll_epiinfo | How would you rate the usefulness of **EpiInfo**? |  | Select one: 1- not useful, 2, 3, 4, 5- very useful |  |  | yes |
| ease_coll_epiinfo | How would you rate the ease of use of **EpiInfo**? |  | Select one: 1- difficult to use, 2, 3, 4, 5- easy to use |  |  | yes |
| improvement_coll_epiinfo | How could **EpiInfo** be improved, if at all? |  | Text |  |  | yes |
| reason_coll_epiinfo | Why was **EpiInfo** chosen over other available tools? |  | Text |  |  | yes |
| users_coll_epiinfo | Who are the main users of **EpiInfo** in your organisation? |  | Select all that apply: Data analyst, Data manager, Data scientist, Epidemiologist, Field coordinator, Field data collectors, Other |  |  | yes |
| users_coll_epiinfo_other | Please specify the other users of **EpiInfo** | selected(${users_coll_epiinfo}, 'other') | Text |  |  |  |
| alerts | Alert management |  | Select one: Yes, No |  |  | yes |
| case_investigation | Case investigation |  | Select one: Yes, No |  |  | yes |
| contact_tracing | Contact tracing |  | Select one: Yes, No |  |  | yes |
| health_promotion | To collect health promotion data |  | Select one: Yes, No |  |  | yes |
| hospital | To collect clinical data |  | Select one: Yes, No |  |  | yes |
| ipc | To conduct IPC assessments |  | Select one: Yes, No |  |  | yes |
| lab | To collect laboratory data |  | Select one: Yes, No |  |  | yes |
| surveillance | To collect surveillance data |  | Select one: Yes, No |  |  | yes |
| trials | To conduct clinical trials |  | Select one: Yes, No |  |  | yes |
| wash | To conduct WASH assessments |  | Select one: Yes, No |  |  | yes |
| other_enihs | Other |  | Select one: Yes, No |  |  | yes |
| other_enihs_purpose | Please specify other use(s) of **eNIHS** | ${other_enihs} = 'yes' | Text |  |  |  |
| rate_coll_enihs | How would you rate the usefulness of **eNIHS**? |  | Select one: 1- not useful, 2, 3, 4, 5- very useful |  |  | yes |
| ease_coll_enihs | How would you rate the ease of use of **eNIHS**? |  | Select one: 1- difficult to use, 2, 3, 4, 5- easy to use |  |  | yes |
| improvement_coll_enihs | How could **eNIHS** be improved, if at all? |  | Text |  |  | yes |
| reason_coll_enihs | Why was **eNIHS** chosen over other available tools? |  | Text |  |  | yes |
| users_coll_enihs | Who are the main users of **eNIHS** in your organisation? |  | Select all that apply: Data analyst, Data manager, Data scientist, Epidemiologist, Field coordinator, Field data collectors, Other |  |  | yes |
| users_coll_enihs_other | Please specify the other users of **eNIHS** | selected(${users_coll_enihs}, 'other') | Text |  |  |  |
| alerts | Alert management |  | Select one: Yes, No |  |  | yes |
| case_investigation | Case investigation |  | Select one: Yes, No |  |  | yes |
| contact_tracing | Contact tracing |  | Select one: Yes, No |  |  | yes |
| health_promotion | To collect health promotion data |  | Select one: Yes, No |  |  | yes |
| hospital | To collect clinical data |  | Select one: Yes, No |  |  | yes |
| ipc | To conduct IPC assessments |  | Select one: Yes, No |  |  | yes |
| lab | To collect laboratory data |  | Select one: Yes, No |  |  | yes |
| surveillance | To collect surveillance data |  | Select one: Yes, No |  |  | yes |
| trials | To conduct clinical trials |  | Select one: Yes, No |  |  | yes |
| wash | To conduct WASH assessments |  | Select one: Yes, No |  |  | yes |
| other_ewars | Other |  | Select one: Yes, No |  |  | yes |
| other_ewars_purpose | Please specify other use(s) of **EWARS** | ${other_ewars} = 'yes' | Text |  |  |  |
| rate_coll_ewars | How would you rate the usefulness of **EWARS**? |  | Select one: 1- not useful, 2, 3, 4, 5- very useful |  |  | yes |
| ease_coll_ewars | How would you rate the ease of use of **EWARS**? |  | Select one: 1- difficult to use, 2, 3, 4, 5- easy to use |  |  | yes |
| improvement_coll_ewars | How could **EWARS** be improved, if at all? |  | Text |  |  | yes |
| reason_coll_ewars | Why was **EWARS** chosen over other available tools? |  | Text |  |  | yes |
| users_coll_ewars | Who are the main users of **EWARS** in your organisation? |  | Select all that apply: Data analyst, Data manager, Data scientist, Epidemiologist, Field coordinator, Field data collectors, Other |  |  | yes |
| users_coll_ewars_other | Please specify the other users of **EWARS** | selected(${users_coll_ewars}, 'other') | Text |  |  |  |
| alerts | Alert management |  | Select one: Yes, No |  |  | yes |
| case_investigation | Case investigation |  | Select one: Yes, No |  |  | yes |
| contact_tracing | Contact tracing |  | Select one: Yes, No |  |  | yes |
| health_promotion | To collect health promotion data |  | Select one: Yes, No |  |  | yes |
| hospital | To collect clinical data |  | Select one: Yes, No |  |  | yes |
| ipc | To conduct IPC assessments |  | Select one: Yes, No |  |  | yes |
| lab | To collect laboratory data |  | Select one: Yes, No |  |  | yes |
| surveillance | To collect surveillance data |  | Select one: Yes, No |  |  | yes |
| trials | To conduct clinical trials |  | Select one: Yes, No |  |  | yes |
| wash | To conduct WASH assessments |  | Select one: Yes, No |  |  | yes |
| other_godata | Other |  | Select one: Yes, No |  |  | yes |
| other_godata_purpose | Please specify other use(s) of **Go.Data** | ${other_godata} = 'yes' | Text |  |  |  |
| rate_coll_godata | How would you rate the usefulness of **Go.Data**? |  | Select one: 1- not useful, 2, 3, 4, 5- very useful |  |  | yes |
| ease_coll_godata | How would you rate the ease of use of **Go.Data**? |  | Select one: 1- difficult to use, 2, 3, 4, 5- easy to use |  |  | yes |
| improvement_coll_godata | How could **Go.Data** be improved, if at all? |  | Text |  |  | yes |
| reason_coll_godata | Why was **Go.Data** chosen over other available tools? |  | Text |  |  | yes |
| users_coll_godata | Who are the main users of **Go.Data** in your organisation? |  | Select all that apply: Data analyst, Data manager, Data scientist, Epidemiologist, Field coordinator, Field data collectors, Other |  |  | yes |
| users_coll_godata_other | Please specify the other users of **Go.Data** | selected(${users_coll_godata}, 'other') | Text |  |  |  |
| alerts | Alert management |  | Select one: Yes, No |  |  | yes |
| case_investigation | Case investigation |  | Select one: Yes, No |  |  | yes |
| contact_tracing | Contact tracing |  | Select one: Yes, No |  |  | yes |
| health_promotion | To collect health promotion data |  | Select one: Yes, No |  |  | yes |
| hospital | To collect clinical data |  | Select one: Yes, No |  |  | yes |
| ipc | To conduct IPC assessments |  | Select one: Yes, No |  |  | yes |
| lab | To collect laboratory data |  | Select one: Yes, No |  |  | yes |
| surveillance | To collect surveillance data |  | Select one: Yes, No |  |  | yes |
| trials | To conduct clinical trials |  | Select one: Yes, No |  |  | yes |
| wash | To conduct WASH assessments |  | Select one: Yes, No |  |  | yes |
| other_kobo | Other |  | Select one: Yes, No |  |  | yes |
| other_kobo_purpose | Please specify other use(s) of **KoBoCollect** | ${other_kobo} = 'yes' | Text |  |  |  |
| rate_coll_kobo | How would you rate the usefulness of **KoBoCollect**? |  | Select one: 1- not useful, 2, 3, 4, 5- very useful |  |  | yes |
| ease_coll_kobo | How would you rate the ease of use of **KoBoCollect**? |  | Select one: 1- difficult to use, 2, 3, 4, 5- easy to use |  |  | yes |
| improvement_coll_kobo | How could **KoBoCollect** be improved, if at all? |  | Text |  |  | yes |
| reason_coll_kobo | Why was **KoBoCollect** chosen over other available tools? |  | Text |  |  | yes |
| users_coll_kobo | Who are the main users of **KoBoCollect** in your organisation? |  | Select all that apply: Data analyst, Data manager, Data scientist, Epidemiologist, Field coordinator, Field data collectors, Other |  |  | yes |
| users_coll_kobo_other | Please specify the other users of **KoBoCollect** | selected(${users_coll_kobo}, 'other') | Text |  |  |  |
| alerts | Alert management |  | Select one: Yes, No |  |  | yes |
| case_investigation | Case investigation |  | Select one: Yes, No |  |  | yes |
| contact_tracing | Contact tracing |  | Select one: Yes, No |  |  | yes |
| health_promotion | To collect health promotion data |  | Select one: Yes, No |  |  | yes |
| hospital | To collect clinical data |  | Select one: Yes, No |  |  | yes |
| ipc | To conduct IPC assessments |  | Select one: Yes, No |  |  | yes |
| lab | To collect laboratory data |  | Select one: Yes, No |  |  | yes |
| surveillance | To collect surveillance data |  | Select one: Yes, No |  |  | yes |
| trials | To conduct clinical trials |  | Select one: Yes, No |  |  | yes |
| wash | To conduct WASH assessments |  | Select one: Yes, No |  |  | yes |
| other_magpi | Other |  | Select one: Yes, No |  |  | yes |
| other_magpi_purpose | Please specify other use(s) of **Magpi** | ${other_magpi} = 'yes' | Text |  |  |  |
| rate_coll_magpi | How would you rate the usefulness of **Magpi**? |  | Select one: 1- not useful, 2, 3, 4, 5- very useful |  |  | yes |
| ease_coll_magpi | How would you rate the ease of use of **Magpi**? |  | Select one: 1- difficult to use, 2, 3, 4, 5- easy to use |  |  | yes |
| improvement_coll_magpi | How could **Magpi** be improved, if at all? |  | Text |  |  | yes |
| reason_coll_magpi | Why was **Magpi** chosen over other available tools? |  | Text |  |  | yes |
| users_coll_magpi | Who are the main users of **Magpi** in your organisation? |  | Select all that apply: Data analyst, Data manager, Data scientist, Epidemiologist, Field coordinator, Field data collectors, Other |  |  | yes |
| users_coll_magpi_other | Please specify the other users of **Magpi** | selected(${users_coll_magpi}, 'other') | Text |  |  |  |
| alerts | Alert management |  | Select one: Yes, No |  |  | yes |
| case_investigation | Case investigation |  | Select one: Yes, No |  |  | yes |
| contact_tracing | Contact tracing |  | Select one: Yes, No |  |  | yes |
| health_promotion | To collect health promotion data |  | Select one: Yes, No |  |  | yes |
| hospital | To collect clinical data |  | Select one: Yes, No |  |  | yes |
| ipc | To conduct IPC assessments |  | Select one: Yes, No |  |  | yes |
| lab | To collect laboratory data |  | Select one: Yes, No |  |  | yes |
| surveillance | To collect surveillance data |  | Select one: Yes, No |  |  | yes |
| trials | To conduct clinical trials |  | Select one: Yes, No |  |  | yes |
| wash | To conduct WASH assessments |  | Select one: Yes, No |  |  | yes |
| other_odk | Other |  | Select one: Yes, No |  |  |  |
| other_odk_purpose | Please specify other use(s) of **ODK** | ${other_odk} = 'yes' | Text |  |  |  |
| rate_coll_odk | How would you rate the usefulness of **ODK**? |  | Select one: 1- not useful, 2, 3, 4, 5- very useful |  |  | yes |
| ease_coll_odk | How would you rate the ease of use of **ODK**? |  | Select one: 1- difficult to use, 2, 3, 4, 5- easy to use |  |  | yes |
| improvement_coll_odk | How could **ODK** be improved, if at all? |  | Text |  |  | yes |
| reason_coll_odk | Why was **ODK** chosen over other available tools? |  | Text |  |  | yes |
| users_coll_odk | Who are the main users of **ODK** in your organisation? |  | Select all that apply: Data analyst, Data manager, Data scientist, Epidemiologist, Field coordinator, Field data collectors, Other |  |  | yes |
| users_coll_odk_other | Please specify the other users of **ODK** | selected(${users_coll_odk}, 'other') | Text |  |  |  |
| alerts | Alert management |  | Select one: Yes, No |  |  | yes |
| case_investigation | Case investigation |  | Select one: Yes, No |  |  | yes |
| contact_tracing | Contact tracing |  | Select one: Yes, No |  |  | yes |
| health_promotion | To collect health promotion data |  | Select one: Yes, No |  |  | yes |
| hospital | To collect clinical data |  | Select one: Yes, No |  |  | yes |
| ipc | To conduct IPC assessments |  | Select one: Yes, No |  |  | yes |
| lab | To collect laboratory data |  | Select one: Yes, No |  |  | yes |
| surveillance | To collect surveillance data |  | Select one: Yes, No |  |  | yes |
| trials | To conduct clinical trials |  | Select one: Yes, No |  |  | yes |
| wash | To conduct WASH assessments |  | Select one: Yes, No |  |  | yes |
| other_openmrs | Other |  | Select one: Yes, No |  |  | yes |
| other_openmrs_purpose | Please specify other use(s) of **OpenMRS** | ${other_openmrs} = 'yes' | Text |  |  |  |
| rate_coll_openmrs | How would you rate the usefulness of **OpenMRS**? |  | Select one: 1- not useful, 2, 3, 4, 5- very useful |  |  | yes |
| ease_coll_openmrs | How would you rate the ease of use of **OpenMRS**? |  | Select one: 1- difficult to use, 2, 3, 4, 5- easy to use |  |  | yes |
| improvement_coll_openmrs | How could **OpenMRS** be improved, if at all? |  | Text |  |  | yes |
| reason_coll_openmrs | Why was **OpenMRS** chosen over other available tools? |  | Text |  |  | yes |
| users_coll_openmrs | Who are the main users of **OpenMRS** in your organisation? |  | Select all that apply: Data analyst, Data manager, Data scientist, Epidemiologist, Field coordinator, Field data collectors, Other |  |  | yes |
| users_coll_openmrs_other | Please specify the other users of **OpenMRS** | selected(${users_coll_openmrs}, 'other') | Text |  |  |  |
| alerts | Alert management |  | Select one: Yes, No |  |  | yes |
| case_investigation | Case investigation |  | Select one: Yes, No |  |  | yes |
| contact_tracing | Contact tracing |  | Select one: Yes, No |  |  | yes |
| health_promotion | To collect health promotion data |  | Select one: Yes, No |  |  | yes |
| hospital | To collect clinical data |  | Select one: Yes, No |  |  | yes |
| ipc | To conduct IPC assessments |  | Select one: Yes, No |  |  | yes |
| lab | To collect laboratory data |  | Select one: Yes, No |  |  | yes |
| surveillance | To collect surveillance data |  | Select one: Yes, No |  |  | yes |
| trials | To conduct clinical trials |  | Select one: Yes, No |  |  | yes |
| wash | To conduct WASH assessments |  | Select one: Yes, No |  |  | yes |
| other_rapidpro | Other |  | Select one: Yes, No |  |  | yes |
| other_rapidpro_purpose | Please specify other use(s) of **RapidPro** | ${other_rapidpro} = 'yes' | Text |  |  |  |
| rate_coll_rapidpro | How would you rate the usefulness of **RapidPro**? |  | Select one: 1- not useful, 2, 3, 4, 5- very useful |  |  | yes |
| ease_coll_rapidpro | How would you rate the ease of use of **RapidPro**? |  | Select one: 1- difficult to use, 2, 3, 4, 5- easy to use |  |  | yes |
| improvement_coll_rapidpro | How could **RapidPro** be improved, if at all? |  | Text |  |  | yes |
| reason_coll_rapidpro | Why was **RapidPro** chosen over other available tools? |  | Text |  |  | yes |
| users_coll_rapidpro | Who are the main users of **RapidPro** in your organisation? |  | Select all that apply: Data analyst, Data manager, Data scientist, Epidemiologist, Field coordinator, Field data collectors, Other |  |  | yes |
| users_coll_rapidpro_other | Please specify the other users of **RapidPro** | selected(${users_coll_rapidpro}, 'other') | Text |  |  |  |
| alerts | Alert management |  | Select one: Yes, No |  |  | yes |
| case_investigation | Case investigation |  | Select one: Yes, No |  |  | yes |
| contact_tracing | Contact tracing |  | Select one: Yes, No |  |  | yes |
| health_promotion | To collect health promotion data |  | Select one: Yes, No |  |  | yes |
| hospital | To collect clinical data |  | Select one: Yes, No |  |  | yes |
| ipc | To conduct IPC assessments |  | Select one: Yes, No |  |  | yes |
| lab | To collect laboratory data |  | Select one: Yes, No |  |  | yes |
| surveillance | To collect surveillance data |  | Select one: Yes, No |  |  | yes |
| trials | To conduct clinical trials |  | Select one: Yes, No |  |  | yes |
| wash | To conduct WASH assessments |  | Select one: Yes, No |  |  | yes |
| other_redcap | Other |  | Select one: Yes, No |  |  | yes |
| other_redcap_purpose | Please specify other use(s) of **REDCap** | ${other_redcap} = 'yes' | Text |  |  |  |
| rate_coll_redcap | How would you rate the usefulness of **REDCap**? |  | Select one: 1- not useful, 2, 3, 4, 5- very useful |  |  | yes |
| ease_coll_redcap | How would you rate the ease of use of **REDCap**? |  | Select one: 1- difficult to use, 2, 3, 4, 5- easy to use |  |  | yes |
| improvement_coll_redcap | How could **REDCap** be improved, if at all? |  | Text |  |  | yes |
| reason_coll_redcap | Why was **REDCap** chosen over other available tools? |  | Text |  |  | yes |
| users_coll_redcap | Who are the main users of **REDCap** in your organisation? |  | Select all that apply: Data analyst, Data manager, Data scientist, Epidemiologist, Field coordinator, Field data collectors, Other |  |  | yes |
| users_coll_redcap_other | Please specify the other users of **REDCap** | selected(${users_coll_redcap}, 'other') | Text |  |  |  |
| alerts | Alert management |  | Select one: Yes, No |  |  | yes |
| case_investigation | Case investigation |  | Select one: Yes, No |  |  | yes |
| contact_tracing | Contact tracing |  | Select one: Yes, No |  |  | yes |
| health_promotion | To collect health promotion data |  | Select one: Yes, No |  |  | yes |
| hospital | To collect clinical data |  | Select one: Yes, No |  |  | yes |
| ipc | To conduct IPC assessments |  | Select one: Yes, No |  |  | yes |
| lab | To collect laboratory data |  | Select one: Yes, No |  |  | yes |
| surveillance | To collect surveillance data |  | Select one: Yes, No |  |  | yes |
| trials | To conduct clinical trials |  | Select one: Yes, No |  |  | yes |
| wash | To conduct WASH assessments |  | Select one: Yes, No |  |  | yes |
| other_sormas | Other |  | Select one: Yes, No |  |  | yes |
| other_sormas_purpose | Please specify other use(s) of **SORMAS** | ${other_sormas} = 'yes' | Text |  |  |  |
| rate_coll_sormas | How would you rate the usefulness of **SORMAS**? |  | Select one: 1- not useful, 2, 3, 4, 5- very useful |  |  | yes |
| ease_coll_sormas | How would you rate the ease of use of **SORMAS**? |  | Select one: 1- difficult to use, 2, 3, 4, 5- easy to use |  |  | yes |
| improvement_coll_sormas | How could **SORMAS** be improved, if at all? |  | Text |  |  | yes |
| reason_coll_sormas | Why was **SORMAS** chosen over other available tools? |  | Text |  |  | yes |
| users_coll_sormas | Who are the main users of **SORMAS** in your organisation? |  | Select all that apply: Data analyst, Data manager, Data scientist, Epidemiologist, Field coordinator, Field data collectors, Other |  |  | yes |
| users_coll_sormas_other | Please specify the other users of **SORMAS** | selected(${users_coll_sormas}, 'other') | Text |  |  |  |
| alerts | Alert management |  | Select one: Yes, No |  |  | yes |
| case_investigation | Case investigation |  | Select one: Yes, No |  |  | yes |
| contact_tracing | Contact tracing |  | Select one: Yes, No |  |  | yes |
| health_promotion | To collect health promotion data |  | Select one: Yes, No |  |  | yes |
| hospital | To collect clinical data |  | Select one: Yes, No |  |  | yes |
| ipc | To conduct IPC assessments |  | Select one: Yes, No |  |  | yes |
| lab | To collect laboratory data |  | Select one: Yes, No |  |  | yes |
| surveillance | To collect surveillance data |  | Select one: Yes, No |  |  | yes |
| trials | To conduct clinical trials |  | Select one: Yes, No |  |  | yes |
| wash | To conduct WASH assessments |  | Select one: Yes, No |  |  | yes |
| other_surveycto | Other |  | Select one: Yes, No |  |  | yes |
| other_surveycto_purpose | Please specify other use(s) of **SurveyCTO** | ${other_surveycto} = 'yes' | Text |  |  |  |
| rate_coll_surveycto | How would you rate the usefulness of **SurveyCTO**? |  | Select one: 1- not useful, 2, 3, 4, 5- very useful |  |  | yes |
| ease_coll_surveycto | How would you rate the ease of use of **SurveyCTO**? |  | Select one: 1- difficult to use, 2, 3, 4, 5- easy to use |  |  | yes |
| improvement_coll_surveycto | How could **SurveyCTO** be improved, if at all? |  | Text |  |  | yes |
| reason_coll_surveycto | Why was **SurveyCTO** chosen over other available tools? |  | Text |  |  | yes |
| users_coll_surveycto | Who are the main users of **SurveyCTO** in your organisation? |  | Select all that apply: Data analyst, Data manager, Data scientist, Epidemiologist, Field coordinator, Field data collectors, Other |  |  | yes |
| users_coll_surveycto_other | Please specify the other users of **SurveyCTO** | selected(${users_coll_surveycto}, 'other') | Text |  |  |  |
| alerts | Alert management |  | Select one: Yes, No |  |  | yes |
| case_investigation | Case investigation |  | Select one: Yes, No |  |  | yes |
| contact_tracing | Contact tracing |  | Select one: Yes, No |  |  | yes |
| health_promotion | To collect health promotion data |  | Select one: Yes, No |  |  | yes |
| hospital | To collect clinical data |  | Select one: Yes, No |  |  | yes |
| ipc | To conduct IPC assessments |  | Select one: Yes, No |  |  | yes |
| lab | To collect laboratory data |  | Select one: Yes, No |  |  | yes |
| surveillance | To collect surveillance data |  | Select one: Yes, No |  |  | yes |
| trials | To conduct clinical trials |  | Select one: Yes, No |  |  | yes |
| wash | To conduct WASH assessments |  | Select one: Yes, No |  |  | yes |
| other_coll | Other |  | Select one: Yes, No |  |  | yes |
| other_other_purpose | Please specify other use(s) of ${other_collect_name} | ${other_coll} = 'yes' | Text |  |  |  |
| rate_coll_other | How would you rate the usefulness of ${other_collect_name}? |  | Select one: 1- not useful, 2, 3, 4, 5- very useful |  |  | yes |
| ease_coll_other | How would you rate the ease of use of ${other_collect_name}? |  | Select one: 1- difficult to use, 2, 3, 4, 5- easy to use |  |  | yes |
| improvement_coll_other | How could ${other_collect_name} be improved, if at all? |  | Text |  |  | yes |
| reason_coll_other | Why was ${other_collect_name} chosen over other available tools? |  | Text |  |  | yes |
| users_coll_other | Who are the main users of ${other_collect_name} in your organisation? |  | Select all that apply: Data analyst, Data manager, Data scientist, Epidemiologist, Field coordinator, Field data collectors, Other |  |  | yes |
| users_coll_other_other | Please specify the other users of ${other_collect_name} | selected(${users_coll_other}, 'other') | Text |  |  |  |
| management_tool | Which electronic **DATA MANAGEMENT** tool(s) have you used when responding to outbreaks? | selected(${tool}, 'data_management') | Select all that apply: Microsoft Access, EpiData, Excel, MySQL, Other |  |  | yes |
| other_mgmt | Please specify the name of the electronic **DATA MANAGEMENT** tool used | selected(${management_tool}, 'other') | Text |  |  | yes |
| cleaning | Data cleaning |  | Select one: Yes, No |  |  | yes |
| management | Data management |  | Select one: Yes, No |  |  | yes |
| survey | Survey creation |  | Select one: Yes, No |  |  | yes |
| other_access | Other |  | Select one: Yes, No |  |  | yes |
| other_purpose_mgmt_access | Please specify the other use(s) of **Microsoft Access** | ${other_access} ='yes' | Text |  |  |  |
| rate_mgmt_access | How would you rate the usefulness of **Microsoft Access**? |  | Select one: 1- not useful, 2, 3, 4, 5- very useful |  |  | yes |
| ease_mgmt_access | How would you rate the ease of use of **Microsoft Access**? |  | Select one: 1- difficult to use, 2, 3, 4, 5- easy to use |  |  | yes |
| improvement_data_mgmt_access | How could **Microsoft Access** be improved, if at all? |  | Text |  |  | yes |
| reason_mgmt_access | Why was **Microsoft Access** chosen over other available data management tools? |  | Text |  |  | yes |
| users_mgmt_access | Who are the main users of **Microsoft Access** in your organisation? |  | Select all that apply: Data analyst, Data manager, Data scientist, Epidemiologist, Field coordinator, Field data collectors, Other |  |  | yes |
| users_mgmt_access_other | Please specify the other users of **Microsoft Access** | selected(${users_mgmt_access}, 'other') | Text |  |  |  |
| cleaning | Data cleaning |  | Select one: Yes, No |  |  | yes |
| management | Data management |  | Select one: Yes, No |  |  | yes |
| survey | Survey creation |  | Select one: Yes, No |  |  | yes |
| other_epidata | Other |  | Select one: Yes, No |  |  | yes |
| other_purpose_mgmt_epidata | Please specify the other use(s) of **EpiData** | ${other_epidata} ='yes' | Text |  |  |  |
| rate_mgmt_epidata | How would you rate the usefulness of **EpiData**? |  | Select one: 1- not useful, 2, 3, 4, 5- very useful |  |  | yes |
| ease_mgmt_epidata | How would you rate the ease of use of **EpiData**? |  | Select one: 1- difficult to use, 2, 3, 4, 5- easy to use |  |  | yes |
| improvement_data_mgmt_epidata | How could **EpiData** be improved, if at all? |  | Text |  |  | yes |
| reason_mgmt_epidata | Why was **EpiData** chosen over other available data management tools? |  | Text |  |  | yes |
| users_mgmt_epidata | Who are the main users of **EpiData** in your organisation? |  | Select all that apply: Data analyst, Data manager, Data scientist, Epidemiologist, Field coordinator, Field data collectors, Other |  |  | yes |
| users_mgmt_epidata_other | Please specify the other users of **EpiData** | selected(${users_mgmt_epidata}, 'other') | Text |  |  |  |
| cleaning | Data cleaning |  | Select one: Yes, No |  |  | yes |
| management | Data management |  | Select one: Yes, No |  |  | yes |
| survey | Survey creation |  | Select one: Yes, No |  |  | yes |
| other_excel | Other |  | Select one: Yes, No |  |  | yes |
| other_purpose_mgmt_epidata | Please specify the other use(s) of **Excel** | ${other_excel} ='yes' | Text |  |  |  |
| rate_mgmt_excel | How would you rate the usefulness of **Excel**? |  | Select one: 1- not useful, 2, 3, 4, 5- very useful |  |  | yes |
| ease_mgmt_excel | How would you rate the ease of use of **Excel**? |  | Select one: 1- difficult to use, 2, 3, 4, 5- easy to use |  |  | yes |
| improvement_data_mgmt_excel | How could **Excel** be improved, if at all? |  | Text |  |  | yes |
| reason_mgmt_excel | Why was **Excel** chosen over other available data management tools? |  | Text |  |  | yes |
| users_mgmt_excel | Who are the main users of **Excel** in your organisation? |  | Select all that apply: Data analyst, Data manager, Data scientist, Epidemiologist, Field coordinator, Field data collectors, Other |  |  | yes |
| users_mgmt_excel_other | Please specify the other users of **Excel** | selected(${users_mgmt_excel}, 'other') | Text |  |  |  |
| cleaning | Data cleaning |  | Select one: Yes, No |  |  | yes |
| management | Data management |  | Select one: Yes, No |  |  | yes |
| survey | Survey creation |  | Select one: Yes, No |  |  | yes |
| other_mysql | Other |  | Select one: Yes, No |  |  | yes |
| other_purpose_mgmt_mysql | Please specify the other use(s) of **MySQL** | ${other_mysql} ='yes' | Text |  |  |  |
| rate_mgmt_mysql | How would you rate the usefulness of **MySQL**? |  | Select one: 1- not useful, 2, 3, 4, 5- very useful |  |  | yes |
| ease_mgmt_mysql | How would you rate the ease of use of **MySQL**? |  | Select one: 1- difficult to use, 2, 3, 4, 5- easy to use |  |  | yes |
| improvement_data_mgmt_mysql | How could **MySQL** be improved, if at all? |  | Text |  |  | yes |
| reason_mgmt_mysql | Why was **MySQL** chosen over other available data management tools? |  | Text |  |  | yes |
| users_mgmt_mysql | Who are the main users of **MySQL** in your organisation? |  | Select all that apply: Data analyst, Data manager, Data scientist, Epidemiologist, Field coordinator, Field data collectors, Other |  |  | yes |
| users_mgmt_mysql_other | Please specify the other users of **MySQL** | selected(${users_mgmt_mysql}, 'other') | Text |  |  |  |
| cleaning | Data cleaning |  | Select one: Yes, No |  |  | yes |
| management | Data management |  | Select one: Yes, No |  |  | yes |
| survey | Survey creation |  | Select one: Yes, No |  |  | yes |
| other_mgmt_other | Other |  | Select one: Yes, No |  |  | yes |
| other_purpose_mgmt_other | Please specify the other use(s) of ${other_mgmt} | ${other_mgmt_other} ='yes' | Text |  |  |  |
| rate_mgmt_other | How would you rate the usefulness of ${other_mgmt}? |  | Select one: 1- not useful, 2, 3, 4, 5- very useful |  |  | yes |
| ease_mgmt_other | How would you rate the ease of use of ${other_mgmt}? |  | Select one: 1- difficult to use, 2, 3, 4, 5- easy to use |  |  | yes |
| improvement_data_mgmt_other | How could ${other_mgmt} be improved, if at all? |  | Text |  |  | yes |
| reason_mgmt_other | Why was ${other_mgmt} chosen over other available data management tools? |  | Text |  |  | yes |
| users_mgmt_other | Who are the main users of ${other_mgmt} in your organisation? |  | Select all that apply: Data analyst, Data manager, Data scientist, Epidemiologist, Field coordinator, Field data collectors, Other |  |  | yes |
| users_mgmt_other_other | Please specify the other users of ${other_mgmt} | selected(${users_mgmt_other}, 'other') | Text |  |  |  |
| analysis | Which **DATA ANALYSIS** tool(s) have you used when responding to outbreaks? | selected(${tool}, 'data_analysis') | Select all that apply: ArcGIS, EpiInfo, Excel, QGIS, R, SatScan, SPSS, Stata, Tableau, Other |  |  | yes |
| other_analysis | Please specify the name of the data analysis tool used | selected(${analysis},'other') | Text |  |  |  |
| cleaning | Data cleaning |  | Select one: Yes, No |  |  | yes |
| descriptive | Descriptive analysis |  | Select one: Yes, No |  |  | yes |
| mapping | Mapping |  | Select one: Yes, No |  |  | yes |
| spatial_analysis | Spatial analysis |  | Select one: Yes, No |  |  | yes |
| modelling | Modelling/Forecasting |  | Select one: Yes, No |  |  | yes |
| visualisation | Data visualisation |  | Select one: Yes, No |  |  | yes |
| reporting | Data reporting |  | Select one: Yes, No |  |  | yes |
| dashboard | Dashboard creation |  | Select one: Yes, No |  |  | yes |
| other_data_analysis_arc | Other |  | Select one: Yes, No |  |  | yes |
| other_purpose_data_analysis_arc | Please specify the other purpose of **ArcGIS** | ${other_data_analysis_arc} = 'yes' | Text |  |  |  |
| rate_analysis_arc | How would you rate the usefulness of **ArcGIS**? |  | Select one: 1- not useful, 2, 3, 4, 5- very useful |  |  | yes |
| ease_analysis_arc | How would you rate the ease of use of **ArcGIS**? |  | Select one: 1- difficult to use, 2, 3, 4, 5- easy to use |  |  | yes |
| improvement_data_analysis_arc | How could **ArcGIS** be improved, if at all? |  | Text |  |  | yes |
| reason_data_analysis_arc | Why was **ArcGIS** chosen over other available tools? |  | Text |  |  | yes |
| users_analysis_arc | Who are the main users of **ArcGIS** in your organisation? |  | Select all that apply: Data analyst, Data manager, Data scientist, Epidemiologist, Field coordinator, Field data collectors, Other |  |  | yes |
| users_analysis_arc_other | Please specify the other users of **ArcGIS** | selected(${users_analysis_arc}, 'other') | Text |  |  |  |
| cleaning | Data cleaning |  | Select one: Yes, No |  |  | yes |
| descriptive | Descriptive analysis |  | Select one: Yes, No |  |  | yes |
| mapping | Mapping |  | Select one: Yes, No |  |  | yes |
| spatial_analysis | Spatial analysis |  | Select one: Yes, No |  |  | yes |
| modelling | Modelling/Forecasting |  | Select one: Yes, No |  |  | yes |
| visualisation | Data visualisation |  | Select one: Yes, No |  |  | yes |
| reporting | Data reporting |  | Select one: Yes, No |  |  | yes |
| dashboard | Dashboard creation |  | Select one: Yes, No |  |  | yes |
| other_data_analysis_epiinfo | Other |  | Select one: Yes, No |  |  | yes |
| other_purpose_data_analysis_epiinfo | Please specify the purpose of **EpiInfo** | ${other_data_analysis_epiinfo} = 'yes' | Text |  |  |  |
| rate_analysis_epiinfo | How would you rate the usefulness of **EpiInfo**? |  | Select one: 1- not useful, 2, 3, 4, 5- very useful |  |  | yes |
| ease_analysis_epiinfo | How would you rate the ease of use of **EpiInfo**? |  | Select one: 1- difficult to use, 2, 3, 4, 5- easy to use |  |  | yes |
| improvement_data_analysis_epiinfo | How could **EpiInfo** be improved, if at all? |  | Text |  |  | yes |
| reason_data_analysis_epiinfo | Why was **EpiInfo** chosen over other available tools? |  | Text |  |  | yes |
| users_analysis_epiinfo | Who are the main users of **EpiInfo** in your organisation? |  | Select all that apply: Data analyst, Data manager, Data scientist, Epidemiologist, Field coordinator, Field data collectors, Other |  |  | yes |
| users_analysis_epiinfo_other | Please specify the other users of **EpiInfo** | selected(${users_analysis_epiinfo}, 'other') | Text |  |  |  |
| cleaning | Data cleaning |  | Select one: Yes, No |  |  | yes |
| descriptive | Descriptive analysis |  | Select one: Yes, No |  |  | yes |
| mapping | Mapping |  | Select one: Yes, No |  |  | yes |
| spatial_analysis | Spatial analysis |  | Select one: Yes, No |  |  | yes |
| modelling | Modelling/Forecasting |  | Select one: Yes, No |  |  | yes |
| visualisation | Data visualisation |  | Select one: Yes, No |  |  | yes |
| reporting | Data reporting |  | Select one: Yes, No |  |  | yes |
| dashboard | Dashboard creation |  | Select one: Yes, No |  |  | yes |
| other_data_analysis_excel | Other |  | Select one: Yes, No |  |  | yes |
| other_purpose_data_analysis_excel | Please specify the purpose of **Excel** | ${other_data_analysis_excel} = 'yes' | Text |  |  |  |
| rate_analysis_excel | How would you rate the usefulness of **Excel**? |  | Select one: 1- not useful, 2, 3, 4, 5- very useful |  |  | yes |
| ease_analysis_excel | How would you rate the ease of use of **Excel**? |  | Select one: 1- difficult to use, 2, 3, 4, 5- easy to use |  |  | yes |
| improvement_data_analysis_excel | How could **Excel** be improved, if at all? |  | Text |  |  | yes |
| reason_data_analysis_excel | Why was **Excel** chosen over other available tools? |  | Text |  |  | yes |
| users_analysis_excel | Who are the main users of **Excel** in your organisation? |  | Select all that apply: Data analyst, Data manager, Data scientist, Epidemiologist, Field coordinator, Field data collectors, Other |  |  | yes |
| users_analysis_excel_other | Please specify the other users of **Excel** | selected(${users_analysis_excel}, 'other') | Text |  |  |  |
| cleaning | Data cleaning |  | Select one: Yes, No |  |  | yes |
| descriptive | Descriptive analysis |  | Select one: Yes, No |  |  | yes |
| mapping | Mapping |  | Select one: Yes, No |  |  | yes |
| spatial_analysis | Spatial analysis |  | Select one: Yes, No |  |  | yes |
| modelling | Modelling/Forecasting |  | Select one: Yes, No |  |  | yes |
| visualisation | Data visualisation |  | Select one: Yes, No |  |  | yes |
| reporting | Data reporting |  | Select one: Yes, No |  |  | yes |
| dashboard | Dashboard creation |  | Select one: Yes, No |  |  | yes |
| other_data_analysis_qgis | Other |  | Select one: Yes, No |  |  | yes |
| other_purpose_data_analysis_qgis | Please specify the purpose of **QGIS** | ${other_data_analysis_qgis} = 'yes' | Text |  |  |  |
| rate_analysis_qgis | How would you rate the usefulness of **QGIS**? |  | Select one: 1- not useful, 2, 3, 4, 5- very useful |  |  | yes |
| ease_analysis_qgis | How would you rate the ease of use of **QGIS**? |  | Select one: 1- difficult to use, 2, 3, 4, 5- easy to use |  |  | yes |
| improvement_data_analysis_qgis | How could **QGIS** be improved, if at all? |  | Text |  |  | yes |
| reason_data_analysis_qgis | Why was **QGIS** chosen over other available tools? |  | Text |  |  | yes |
| users_analysis_qgis | Who are the main users of **QGIS** in your organisation? |  | Select all that apply: Data analyst, Data manager, Data scientist, Epidemiologist, Field coordinator, Field data collectors, Other |  |  | yes |
| users_analysis_qgis_other | Please specify the other users of **QGIS** | selected(${users_analysis_qgis}, 'other') | Text |  |  |  |
| cleaning | Data cleaning |  | Select one: Yes, No |  |  | yes |
| descriptive | Descriptive analysis |  | Select one: Yes, No |  |  | yes |
| mapping | Mapping |  | Select one: Yes, No |  |  | yes |
| spatial_analysis | Spatial analysis |  | Select one: Yes, No |  |  | yes |
| modelling | Modelling/Forecasting |  | Select one: Yes, No |  |  | yes |
| visualisation | Data visualisation |  | Select one: Yes, No |  |  | yes |
| reporting | Data reporting |  | Select one: Yes, No |  |  | yes |
| dashboard | Dashboard creation |  | Select one: Yes, No |  |  | yes |
| other_data_analysis_r | Other |  | Select one: Yes, No |  |  | yes |
| other_purpose_data_analysis_r | Please specify the purpose of **R** | ${other_data_analysis_r} = 'yes' | Text |  |  |  |
| rate_analysis_r | How would you rate the usefulness of **R**? |  | Select one: 1- not useful, 2, 3, 4, 5- very useful |  |  | yes |
| ease_analysis_r | How would you rate the ease of use of **R**? |  | Select one: 1- difficult to use, 2, 3, 4, 5- easy to use |  |  | yes |
| improvement_data_analysis_r | How could **R** be improved, if at all? |  | Text |  |  | yes |
| reason_data_analysis_r | Why was **R** chosen over other available tools? |  | Text |  |  | yes |
| users_analysis_r | Who are the main users of **R** in your organisation? |  | Select all that apply: Data analyst, Data manager, Data scientist, Epidemiologist, Field coordinator, Field data collectors, Other |  |  | yes |
| users_analysis_r_other | Please specify the other users of **R** | selected(${users_analysis_r}, 'other') | Text |  |  |  |
| cleaning | Data cleaning |  | Select one: Yes, No |  |  | yes |
| descriptive | Descriptive analysis |  | Select one: Yes, No |  |  | yes |
| mapping | Mapping |  | Select one: Yes, No |  |  | yes |
| spatial_analysis | Spatial analysis |  | Select one: Yes, No |  |  | yes |
| modelling | Modelling/Forecasting |  | Select one: Yes, No |  |  | yes |
| visualisation | Data visualisation |  | Select one: Yes, No |  |  | yes |
| reporting | Data reporting |  | Select one: Yes, No |  |  | yes |
| dashboard | Dashboard creation |  | Select one: Yes, No |  |  | yes |
| other_data_analysis_spss | Other |  | Select one: Yes, No |  |  | yes |
| other_purpose_data_analysis_spps | Please specify the purpose of **SPSS** | ${other_data_analysis_spss} = 'yes' | Text |  |  |  |
| rate_analysis_spss | How would you rate the usefulness of **SPSS**? |  | Select one: 1- not useful, 2, 3, 4, 5- very useful |  |  | yes |
| ease_analysis_spss | How would you rate the ease of use of **SPSS**? |  | Select one: 1- difficult to use, 2, 3, 4, 5- easy to use |  |  | yes |
| improvement_data_analysis_spss | How could **SPSS** be improved, if at all? |  | Text |  |  | yes |
| reason_data_analysis_spss | Why was **SPSS** chosen over other available tools? |  | Text |  |  | yes |
| users_analysis_spss | Who are the main users of **SPSS** in your organisation? |  | Select all that apply: Data analyst, Data manager, Data scientist, Epidemiologist, Field coordinator, Field data collectors, Other |  |  | yes |
| users_analysis_spss_other | Please specify the other users of **SPSS** | selected(${users_analysis_spss}, 'other') | Text |  |  |  |
| cleaning | Data cleaning |  | Select one: Yes, No |  |  | yes |
| descriptive | Descriptive analysis |  | Select one: Yes, No |  |  | yes |
| mapping | Mapping |  | Select one: Yes, No |  |  | yes |
| spatial_analysis | Spatial analysis |  | Select one: Yes, No |  |  | yes |
| modelling | Modelling/Forecasting |  | Select one: Yes, No |  |  | yes |
| visualisation | Data visualisation |  | Select one: Yes, No |  |  | yes |
| reporting | Data reporting |  | Select one: Yes, No |  |  | yes |
| dashboard | Dashboard creation |  | Select one: Yes, No |  |  | yes |
| other_data_analysis_stata | Other |  | Select one: Yes, No |  |  | yes |
| other_purpose_data_analysis_stata | Please specify the purpose of **Stata** | ${other_data_analysis_stata} = 'yes' | Text |  |  |  |
| rate_analysis_stata | How would you rate the usefulness of **Stata**? |  | Select one: 1- not useful, 2, 3, 4, 5- very useful |  |  | yes |
| ease_analysis_stata | How would you rate the ease of use of **Stata**? |  | Select one: 1- difficult to use, 2, 3, 4, 5- easy to use |  |  | yes |
| improvement_data_analysis_stata | How could **Stata** be improved, if at all? |  | Text |  |  | yes |
| reason_data_analysis_stata | Why was **Stata** chosen over other available tools? |  | Text |  |  | yes |
| users_analysis_stata | Who are the main users of **Stata** in your organisation? |  | Select all that apply: Data analyst, Data manager, Data scientist, Epidemiologist, Field coordinator, Field data collectors, Other |  |  | yes |
| users_analysis_stata_other | Please specify the other users of **Stata** | selected(${users_analysis_stata}, 'other') | Text |  |  |  |
| cleaning | Data cleaning |  | Select one: Yes, No |  |  | yes |
| descriptive | Descriptive analysis |  | Select one: Yes, No |  |  | yes |
| mapping | Mapping |  | Select one: Yes, No |  |  | yes |
| spatial_analysis | Spatial analysis |  | Select one: Yes, No |  |  | yes |
| modelling | Modelling/Forecasting |  | Select one: Yes, No |  |  | yes |
| visualisation | Data visualisation |  | Select one: Yes, No |  |  | yes |
| reporting | Data reporting |  | Select one: Yes, No |  |  | yes |
| dashboard | Dashboard creation |  | Select one: Yes, No |  |  | yes |
| other_data_analysis_satscan | Other |  | Select one: Yes, No |  |  | yes |
| other_purpose_data_analysis_satscan | Please specify the purpose of **SatScan** | ${other_data_analysis_satscan} = 'yes' | Text |  |  |  |
| rate_analysis_satscan | How would you rate the usefulness of **SatScan**? |  | Select one: 1- not useful, 2, 3, 4, 5- very useful |  |  | yes |
| ease_analysis_satscan | How would you rate the ease of use of **SatScan**? |  | Select one: 1- difficult to use, 2, 3, 4, 5- easy to use |  |  | yes |
| improvement_data_analysis_satscan | How could **SatScan** be improved, if at all? |  | Text |  |  | yes |
| reason_data_analysis_satscan | Why was **SatScan** chosen over other available tools? |  | Text |  |  | yes |
| users_analysis_satscan | Who are the main users of **SatScan** in your organisation? |  | Select all that apply: Data analyst, Data manager, Data scientist, Epidemiologist, Field coordinator, Field data collectors, Other |  |  | yes |
| users_analysis_satscan_other | Please specify the other users of **SatScan** | selected(${users_analysis_satscan}, 'other') | Text |  |  |  |
| cleaning | Data cleaning |  | Select one: Yes, No |  |  | yes |
| descriptive | Descriptive analysis |  | Select one: Yes, No |  |  | yes |
| mapping | Mapping |  | Select one: Yes, No |  |  | yes |
| spatial_analysis | Spatial analysis |  | Select one: Yes, No |  |  | yes |
| modelling | Modelling/Forecasting |  | Select one: Yes, No |  |  | yes |
| visualisation | Data visualisation |  | Select one: Yes, No |  |  | yes |
| reporting | Data reporting |  | Select one: Yes, No |  |  | yes |
| dashboard | Dashboard creation |  | Select one: Yes, No |  |  | yes |
| other_data_analysis_tableau | Other |  | Select one: Yes, No |  |  | yes |
| other_purpose_data_analysis_tableau | Please specify the purpose of **Tableau** | ${other_data_analysis_tableau} = 'yes' | Text |  |  |  |
| rate_analysis_tableau | How would you rate the usefulness of **Tableau**? |  | Select one: 1- not useful, 2, 3, 4, 5- very useful |  |  | yes |
| ease_analysis_tableau | How would you rate the ease of use of **Tableau**? |  | Select one: 1- difficult to use, 2, 3, 4, 5- easy to use |  |  | yes |
| improvement_data_analysis_tableau | How could **Tableau** be improved, if at all? |  | Text |  |  | yes |
| reason_data_analysis_tableau | Why was **Tableau** chosen over other available tools? |  | Text |  |  | yes |
| users_analysis_tableau | Who are the main users of **Tableau** in your organisation? |  | Select all that apply: Data analyst, Data manager, Data scientist, Epidemiologist, Field coordinator, Field data collectors, Other |  |  | yes |
| users_analysis_tableau_other | Please specify the other users of **Tableau** | selected(${users_analysis_tableau}, 'other') | Text |  |  |  |
| cleaning | Data cleaning |  | Select one: Yes, No |  |  | yes |
| descriptive | Descriptive analysis |  | Select one: Yes, No |  |  | yes |
| mapping | Mapping |  | Select one: Yes, No |  |  | yes |
| spatial_analysis | Spatial analysis |  | Select one: Yes, No |  |  | yes |
| modelling | Modelling/Forecasting |  | Select one: Yes, No |  |  | yes |
| visualisation | Data visualisation |  | Select one: Yes, No |  |  | yes |
| reporting | Data reporting |  | Select one: Yes, No |  |  | yes |
| dashboard | Dashboard creation |  | Select one: Yes, No |  |  | yes |
| other_data_analysis_other | Other |  | Select one: Yes, No |  |  | yes |
| other_purpose_data_analysis_other | Please specify the purpose of ${other_analysis} | ${other_data_analysis_other} = 'yes' | Text |  |  |  |
| rate_analysis_other | How would you rate the usefulness of ${other_analysis}? |  | Select one: 1- not useful, 2, 3, 4, 5- very useful |  |  | yes |
| ease_analysis_other | How would you rate the ease of use of ${other_analysis}? |  | Select one: 1- difficult to use, 2, 3, 4, 5- easy to use |  |  | yes |
| improvement_data_analysis_other | How could ${other_analysis} be improved, if at all? |  | Text |  |  | yes |
| reason_data_analysis_other | Why was ${other_analysis} chosen over other available tools? |  | Text |  |  | yes |
| users_analysis_other | Who are the main users of ${other_analysis} in your organisation? |  | Select all that apply: Data analyst, Data manager, Data scientist, Epidemiologist, Field coordinator, Field data collectors, Other |  |  | yes |
| users_analysis_other_other | Please specify the other users of ${other_analysis} | selected(${users_analysis_other}, 'other') | Text |  |  |  |
| challenges_coll | Please rank the following challenges when collecting data during an outbreak? |  |  |  |  | yes |
| other_challenges | Please specify any other data collection challenges you encounter during outbreaks |  | Text |  |  |  |
| useful_tool_collect | Please rank the usefulness of the following for data collection in an outbreak or emergency setting? Please rank from top to bottom, such that your first option equates to the item with the most utility |  |  |  |  | yes |
| other_useful_tool | Please specify what kind of tool would be helpful | ${useful_tool_collect} = 'other' | Text |  |  |  |
| other_comments | Do you have any additional comments to provide on data collection, management or analysis tools during outbreaks? |  | Text |  |  |  |
