# Supplementary material for: Electronic data collection, management and analysis tools used for outbreak response in low- and middle-income countries: a systematic review and stakeholder survey
Source: BMC Public Health. 2021 Sep 25;21:1741. doi: 10.1186/s12889-021-11790-w (PMC8464108; doi:10.1186/s12889-021-11790-w)
Supplement: Supplementary file 5 — Additional file 5. Number and percentage of respondents to the stakeholder survey per responding organisation. Table shows the number and percentages of respondents per organisation that responded to the stakeholder survey. [file 12889_2021_11790_MOESM5_ESM.docx]

Number and percentage of respondents to the stakeholder survey per responding organisation

| **Organisation** | **No. of respondents** | **% of respondents** |
| --- | --- | --- |
| AMREF Health Africa | 1 | 2.44 |
| Bayero University Kano | 1 | 2.44 |
| CDC | 2 | 4.88 |
| EMPHNET | 1 | 2.44 |
| Erasmus Medical Center | 1 | 2.44 |
| Institute of Tropical Medicine | 1 | 2.44 |
| Maryland Global Initiative | 1 | 2.44 |
| Medical Research Council Unit at London school of Hygiene | 1 | 2.44 |
| Ministry of Health | 4 | 9.76 |
| MSF | 9 | 21.95 |
| NCDC | 1 | 2.44 |
| National Primary Health Care Development Agency (Nigeria) | 1 | 2.44 |
| Public Health Agency of Canada | 1 | 2.44 |
| RedCross | 2 | 4.88 |
| Samaritan’s Purse International Relief | 1 | 2.44 |
| Save the Children | 4 | 9.76 |
| WHO | 9 | 21.95 |
| **Total** | **41** | **100** |
